# NOX4-derived oxidative DNA damage impairs thyroid differentiation through an epigenetic mechanism in BRAF-mutated radioactive iodine refractory papillary thyroid cancer cells

**DOI:** 10.7150/ijbs.123980

**Published:** 2026-01-15

**Authors:** Mickaëlle Radom, Camille Buffet, Juliana Cazarin, Marylin Harinquet, Caroline Coelho de Faria, Floriane Brayé, Catline Nobre, Marine Aglave, Yasmina Mesloub, Thibault Dayris, Nathalie Droin, Karine Godefroy, Mohamed-Amine Bani, Abir Al Ghuzlan, Sophie Leboulleux, Livia Lamartina, Corinne Dupuy

**Affiliations:** 1 UMR 9019 CNRS, Gustave Roussy, Université Paris-Saclay, Villejuif, France.; 2 Sorbonne Université, Service de Pathologies Thyroïdiennes et Tumeurs endocrines, Groupe de Recherche Clinique n°16 Tumeurs Thyroïdiennes, AP-HP, Hôpital Pitié-Salpêtrière, Paris, France.; 3 Instituto de Biofísica Carlos Chagas Filho, Universidade Federal do Rio de Janeiro, 21941-170, RJ, Brazil.; 4 INSERM U981, Gustave Roussy, Université Paris-Saclay, Villejuif, France.; 5 Bioinformatics Plateform, Gustave Roussy, Villejuif, France.; 6 AMMICa Platform, INSERM US23, CNRS UAR 3655, Gustave Roussy, Villejuif, France.; 7 Cancer Medical Pathology and Biology Department, Gustave Roussy, Villejuif, France.; 8 Department of Endocrinology and Diabetology, Hôpitaux Universitaires de Genève, Geneve, Switzerland.; 9 Cancer Medicine Department, Gustave Roussy, Université Paris Saclay, Villejuif, France.

**Keywords:** NADPH oxidase, ROS, Oxidative DNA damage, BRAF^V600E^, Radioiodine refractory thyroid cancer.

## Abstract

Radioiodine (RAI) therapy, used for treating differentiated thyroid cancers (DTCs), hinges on the functional expression of the sodium-iodide symporter (NIS). However, up to 60% of papillary thyroid carcinomas, the most common DTC subtype, harbor BRAF^V600E^ mutations, which are strongly associated with reduced NIS expression, impaired RAI uptake, and poor differentiation scores. For patients with RAI-refractory, a promising therapeutic strategy is to restore RAI sensitivity by inducing tumor redifferentiation. Here, we demonstrate that NOX4-derived reactive oxygen species (ROS) contribute to NIS repression in BRAF^V600E^-mutated thyroid cancer cells. Particularly, NOX4-generated oxidative DNA damage recruits DNA repair proteins, including OGG1 and MSH2/MSH6 proteins, which in cooperation with DNMT1, convert these lesions into transcription-blocking events. This mechanism prevents key thyroid differentiation transcription factors, PAX8 and NKX2.1, from accessing their chromatin binding sites, thereby silencing NIS expression. Importantly, combining inhibition of the MAPK pathway, which regulates MSH2/MSH6 and DNMT1 expression, and the TGF-β1 pathway, which controls NOX4 expression, restores PAX8 and NKX2.1 chromatin occupancy. Compared to normal tissue an increased expression of NOX4, OGG1, MSH2/MSH6 proteins and phospho-Smad3 was found in RAI Refractory BRAF^V600E^ mutated tumors. Collectively, our findings reveal a mechanistic basis for NOX4's role in thyroid dedifferentiation.

## Introduction

Radioiodine therapy (RAI) relies on the capacity of thyroid cells to uptake and concentrate iodide. The sodium iodide symporter (NIS), situated in the basolateral membrane, mediates the active transport of iodide from the bloodstream into thyroid cells. The iodide is then processed by the unique iodide-metabolizing machinery involving, in particular, the thyroid peroxidase (TPO), a key thyroid enzyme incorporating iodide into thyroglobulin (Tg) during thyroid hormone synthesis. This machinery, which is controlled by TSH (Thyroid Stimulating Hormone), concentrates iodine into the cells, and has a significant impact on the efficacy of radioiodine 1. Dedifferentiation correlates with a decrease or, in some cases, a complete loss of expression of thyroid-specific genes (such as *SLC5A5* (encoding NIS), *TPO, TG, TSHR* (encoding TSH receptor)) and thyroid transcription factor genes (like *PAX8* and *NKX2.1* (TTF1). These last two genes play a critical role in regulating gene transcription activity in thyroid follicular cells 2.

A treatment approach for RAI-refractory patients is to re-enhance RAI uptake or re-differentiate tumors 1. Indeed, restoring RAI uptake is the initial step of redifferentiation.

Most differentiated thyroid cancers (DTC) are papillary types (PTC), accounting for 80% of cases. These PTCs are often marked by the presence of the BRAF^V600E^ mutation in 45% to 60% of cases. This mutation is linked with aggressive tumor growth, low gene expression levels related to iodide metabolism, and low tumor differentiation scores 3 - all culminating in resistance to radioiodine (RAI) treatment. The precise mechanism behind this phenomenon remains obscure. BRAF is a potent activator of the MEK/MAP kinase pathway. Interestingly, in a designed mouse model, it was observed that inhibiting mutated BRAF or MEK resulted in reactivating NIS expression and thyroid tumor iodide uptake 4. Nevertheless, the results varied in human studies 5. Hence, deciphering the mechanisms behind the lowered expression of NIS and other thyroid function-related genes is crucial for establishing new molecular targets and developing novel treatment approaches.

Numerous studies have conclusively demonstrated that reactive oxygen species (ROS), via a variety of redox reactions, regulate cellular functions including gene expressions. In cells, NADPH oxidases (NOXs/DUOXs), which are membrane-bound complexes, are totally devoted to ROS production 6. We previously demonstrated that ROS derived from NOX4 contributes to the repression of *SLC5A5* at the transcriptional level 7. The expression level of NOX4 is significantly elevated in both human and murine BRAF-mutated thyroid tumors and is inversely correlated with thyroid differentiation. This suggests that genes involved in thyroid differentiation might be suppressed by a mechanism controlled by ROS derived from NOX4 7.

The reversibility of the *SLC5A5* gene's extinction by NOX4 suggests its contribution to an epigenetic mechanism, including gene methylation by DNA methyltransferases (DNMTs). Recent findings have revealed a connection between oxidative damage repair by DNA mismatch repair (MSH2-MSH6) proteins and transcriptional inhibition by DNMT1 at DNA damage sites 8. In this study, we demonstrate that NOX4 leads to oxidative DNA damage in BRAF^V600E^-mutated thyroid cancer cells. These damages are transformed into transcription-blocking injuries by OGG1 and MMR proteins, which collaborate with DNMT1 and impede the binding of PAX8 and NKX2.1 (TTF1) to the chromatin, these are two key transcription factors involved in thyroid differentiation. Co-inhibition of the MAPK pathway and NOX4 enhances the recruitment of these two transcription factors to the chromatin. We also show that NOX4, OGG1, MSH2 and MSH6 are upregulated in Radioactive Iodine Refractory (RAIR) BRAF^V600E^-mutated thyroid cancer. Overall, our findings offer new insights into the role of NOX4-derived ROS in the thyroid dedifferentiation process.

## Materials and Methods

### Cell culture

The cell lines BCPAP and 8505C were procured from the DSMZ-German Collection and were maintained at a temperature of 37°C and a CO_2_ level of 5% in DMEM (Dulbecco's Modified Eagle Medium) with 4.5 g/L glucose (Life Technologies), and RPMI-1640 (Life Technologies), respectively. Both mediums were supplemented with 10% (vol/vol) FCS (Life Technologies) and 100 mg/ml solution of penicillin/streptomycin (Life Technologies). All cell lines underwent the authentication procedure STR analysis according to the global standard ANSI/ATCC ASN-0002.1-2021 (2021).

### PDX generation and PDX cell line

The PDX563 cell line was derived from a 76-year-old female with BRAF^V600E^-mutated anaplastic thyroid cancer (ATC). She was initially diagnosed with a 3-cm right papillary thyroid carcinoma with extrathyroidal extension in 2010. Treatment involved a total thyroidectomy followed by the administration of 100 mCi I-131. Post-treatment, the control I-131 whole-body scintigraphy and FDG-PET presented as normal. Under LT4 treatment, her serum thyroglobulin level was 0.8 ng/mL. However, in 2017, the serum thyroglobulin level elevated to 3.3 ng/mL. A CT scan revealed a 13-mm right latero-tracheal mass, a 4-mm lymph node in the left compartment VI, and pulmonary micro-nodules. FDG-PET indicated high uptake in the latero-tracheal mass, and a biopsy confirmed a recurrence of the papillary carcinoma. Following a second treatment with 100 mCi of I-131, no uptake was detected on post-therapy whole-body scintigraphy. By the end of 2018, FDG-PET displayed increased uptake in all lesions and a new uptake in the right arm. A biopsy of the right arm revealed an anaplastic carcinoma of the thyroid with significant macrophage infiltration. The patient was treated with a dabrafenib-trametinib combination and external beam radiation to the neck and the right arm. Unfortunately, despite treatment efforts, the neck tumor continued to grow, leading to the patient's death in 2021.

Fresh tissue biopsy fragments of ATC were implanted into the sub-renal capsule of NOD scid gamma (NSG) mice, acquired from Charles River Laboratories. Xenografts were subsequently propagated subcutaneously from mouse to mouse up to five passages to create a viable tumor bank. A patient-derived cell line was developed from PDX samples. These samples were processed through enzymatic digestion using a tumor dissociation kit (Miltenyi Biotec, #130-095-929,) and mechanical degradation using the gentleMACs^TM^ dissociator. The cells were cultivated using DMEM/F-12+GlutamMAX^TM^ 10% FBS solution (Life Technologies) and 10% enriched solution with 0.4 μg/ml hydrocortisone (Sigma, #H4001), 8.4 ng/ml cholera toxin (Sigma, #C9903), 24 μg/ml adenine (Sigma, #A2786), and 5 μM ROCK inhibitor (Sigma, #Y0503), until a stable proliferation of tumor cells was observed.

### Ethics

The patient participating in the study was fully informed and signed an informed consent form. The MATCH-R trial received approval from the ethics committee at Institut Gustave Roussy, as well as the French National Agency for Medicines and Health Products Safety (ANSM), and is conducted following the Declaration of Helsinki. All animal procedures and studies received approval from the French Ministry of Higher Education, Research, and Innovation (APAFIS#2790-2015112015055793). The MERAIODE study is an investigator-initiated trial sponsored by Gustave Roussy, conducted within the French Endocan-TuThyRef network in approved by ethics committees and national authorities and in accordance with the Declaration of Helsinki. All patients gave their written informed consent.

### Cell treatments

For N-Acetyl-L- cysteine (NAC) (Sigma, #A7250) and Diphenylene iodonium (DPI) (Sigma, #300260) treatments, cells were exposed to indicated doses and times at 37 °C in their medium and washed once with PBS prior to harvesting. For dabrafenib plus trametinib treatment, BCPAP cells were exposed to 100 nM dabrafenib (Selleck, #S2807) and 25 nM trametinib (Selleck, #S2673) for indicated time at 37 °C in DMEM with FCS, 8505C cells were exposed to 100 nM dabrafenib and 5 nM trametinib to indicated time at 37 °C in RPMI with FCS and PDX cells were exposed to 100 nM dabrafenib and 5 nM trametinib to indicated time at 37 °C in DMEM/F12 with FCS. After treatments, cells were washed once with PBS prior to harvesting. For Vactosertib (EW-7197) (Selleck, #S7530) treatment, cells were exposed to 1 µM of EW-7197 for indicated time at 37 °C in their respective medium and washed once with PBS prior to harvesting.

### Tight chromatin fractionation and whole-cell protein extraction

The tight chromatin fractionation protocol was adapted with slight modifications from O'Hagan et al 9. Briefly, cell pellets were suspended in buffer A (10 mM HEPES pH 7.9, 10 mM KCl, 1.5 mM MgCl_2_, 0.34 M sucrose, 10 % glycerol, 1mM DTT, 1 × protease and phosphatase inhibitor cocktail (Sigma, #04693116001 and #04906845001). Triton X-100 was then added to the cell suspension to reach a final concentration of 0.1% and incubated for 5 min on ice before centrifugation for 4 min at 1300 × g, 4 °C. The supernatant, enriched in cytoplasm soluble proteins, was retained for analysis (cytosolic fraction) and the nuclei pellet was washed with buffer A. The pellet was then resuspended in buffer B (3 mM EDTA, 0.2 mM EGTA, 1 mM DTT, 1 × protease, and phosphatase inhibitor cocktail) and incubated for 10 min on ice. After that, the nuclei suspension was centrifuged for 4 min at 1700 × g, 4 °C. The supernatant was discarded and the pellet, representing the chromatin fraction, was washed in buffer B and centrifuged for 1 min at 10,000 g, 4 °C. The chromatin fraction was washed with buffer C (50 mM Tris/HCl pH 8, NaCl 0.45 M, IGEPAL 0.05 %, 1 × protease, and phosphatase inhibitor cocktail) to solubilize proteins weakly bound to chromatin and then centrifuged for 1 min at 10,000 g, 4 °C. The remaining pellet was lysed in TEX buffer (100 mM Tris·HCl pH 7.0 containing 2.5% (wt/vol) SDS, 1 mM EDTA, 1 mM EGTA, 4 M urea, and a mixture of phosphatase and protease inhibitors using Qiashredder (Qiagen, #79654) and referred to as tight chromatin. Whole-cell extracts were prepared from 1/10 of the pellet collected after treatment before beginning the tight chromatin isolation. Vinculin and Lamin B immunoblotting served as cytoplasmic and nuclear controls, respectively.

### siRNA knockdown

Cells were transfected at 50-60% confluence with specific human siRNA against NOX4 (Thermo Scientific, silencer select siRNA, #4392421, s224160), scrambled siRNA control (Thermo Scientific, #4392420 and Horizon Perkin Elmer company, On target plus non-targeting control pool, #D-001810-10-05), siRNA against p22^phox^ (Horizon Perkin Elmer company, on target-plus human CYBA siRNA, #L-011020-02-0005), siRNA against OGG1#1 (Thermo Scientific silencer select siRNA, #4390824, s9836), siRNA against OGG1#2 (Thermo Scientific silencer select siRNA, #4390824, s9837), siRNA against DNMT1 (Horizon Perkin Elmer company on target-plus siRNA pool, #L-004605-00-0005), siRNA against MSH2 (Thermo Scientific, Stealth Select RNAiTM, #1299001), and siRNA against MSH6 (Eurogentec, Target sequences: 5′- CCCUGGCAAACAGAUUAAA-3′, 10) using the INTERFERin transfection reagent (Polyplus-Transfection, # POL101000036) following the manufacturer's protocol.

### Plasmid transfection

BRAF^V600E^ Construct—cDNA was synthesized with Maxima reverse transcriptase (Thermo Scientific, # EP0741) by oligo(dT) priming of total RNA from BCPAP cells. The BRAF^V600E^ ORF was amplified using Phusion Plus DNA polymerase (Thermo Scientific, # F630L) and cloned into pcDNA™3.1/V5-His TOPO^®^ vector (Thermo Scientific, # K480001) following the manufacturer's instructions. Primer sequences used for BRAF^V600E^ amplification were: forward: 5'-CACCATGGCGGCGCTGAGCGGTG-3' and reverse: 5' -TCAGTGGACAGGAAACGCACCATATCC-3'. The construct was verified by sequencing.

Twenty-four hours before the experiment, human thyrocytes from the primary culture (2.5 × 10^5^ cells/well) were seeded in 6-well plates. Cells were transiently transfected following the manufacturer's instructions using 3 μl of X-tremGENE HP DNA transfection reagent (Sigma, #6366236001) and 1 μg of total DNA, which consisted of the BRAF^V600E^/pcDNA3.1 plasmid. Experiments were conducted 48 h post-transfection.

### Protein extraction and Western blotting

The Western blot analysis was conducted using lysates prepared as described previously 11. The membranes were probed with primary antibodies: anti-NOX4 (Abcam, #ab109225, RRID:AB_10861375); anti-p22^phox^ (Santa Cruz Biotechnology, #Sc-130551, RRID:AB_2245805); anti-LaminB1 (Abcam, #ab133741, RRID:AB_2616597); anti-MSH6 (Protein Tech, #A3000 22 A); anti-MSH2 (Abcam, #ab70270); anti-OGG1/2 (G5) (Santa Cruz Biotechnology, #Sc376935); anti-Vinculin (Abcam, #ab130007); anti-DNMT1 (Abcam, #ab13537); anti-DNMT3a (Abcam, #ab2850); anti-DNMT3b (Cell signaling, #695202); anti-PAX8 (Cell signaling, #59019); anti-TTF1 (NKX2.1) (Cell signaling, #123735), anti phospho-p44/42 ERK (Cell signaling, #4370); anti-ERK (Cell signaling, #4696); anti phosphor-MEK1/2 (Cell signaling, #9121); anti-MEK (Cell signaling, #4694); anti-BRAF^V600E^ (Spring, #E19290); anti-phospho-Smad3 (Abcam, #ab515663); anti-Smad3 (Thermo Scientific, #MA515663) or anti-Myc-Tag (Cell signaling, #22765) overnight at 4 °C with constant agitation. They were then washed three times using TBS-T and treated with either goat anti-rabbit IgG-HRP antibody (Southern Biotech RRID: AB_2687483) or goat anti-mouse IgG-HRP antibody (Agilent, #P0447, RRID: AB_2617137) for 45 min at room temperature. Following another series of washes - three times with TBS-T - the proteins were visualized through enhanced chemiluminescence (Thermoscientific, #34577).

### Flow cytometry analysis of 8-oxoG

Cells were fixed in 70% ethanol at -20 °C and washed with PBS before being permeabilized with 0.1% Triton X-100/PBS for 15 min at room temperature. They were then washed with PBS and blocked in 2.5% bovine serum albumin/PBS for 30 min at room temperature. The cells were treated with 50µg/ml RNAse and incubated with anti-8-oxodG (Biotechne/R&D systems #4354-MC-050, 1:50 diluted in 2.5% BSA/PBS) or with mouse IgG2B isotype control (Biotechne/R&D systems #MAB004) for 1 h at 37 °C. The cells were then washed once with 0.2% BSA/PBS and incubated with the secondary antibodies Alexa 488 anti-mouse (Invitrogen, #A11017, RRID: AB_2534084) in 0.2% BSA/PBS for 1 h at 37 °C before propidium iodide staining. Cytofluorimetric acquisition and analysis were conducted on a BD Accuri C6 plus Flow cytometer. After exclusion of the cellular debris by appropriate gating on the SSC versus FSC plots, fluorescence intensity was analysed on channel FL-1 (excitation at 488 nm; emission collected at 533/30 nm) measuring at least 20,000 cells for each sample. The results are reported as the mean fluorescence intensity.

### Nuclear H_2_O_2_ production by FACS analysis

The measurement of nuclear H_2_O_2_ was accomplished using NucPE1 (Nuclear Peroxy Emerald 1) (Med Chem Express, #1404091-23-1) 12. The cells were incubated with 10 µM NucPE1 for 45 min in the dark. After incubation, the cells were washed and analyzed by flow cytometry.

### Genomic DNA extraction

Genomic DNA was extracted using the DNeasy Blood & Tissue kit (Qiagen, #69504). All extraction steps were performed under low-light conditions. Additionally, 50 μM N-tert-butyl-α-phenylnitrone (stock solution: 28 mM in H_2_O; Sigma, #B7263,) was added to each DNeasy Blood & Tissue buffer to protect the DNA from further oxidation.

### RNA extraction, cDNA synthesis, and real-time PCR

Total RNA from cell samples was purified with the Nucleospin RNA II kit (Machery Nagel, #740955-50). RNA yield was determined using a spectrophotometer (NanoDrop Technologies, Wilmington, USA). DNase-treated RNA (1 µg) was reverse-transcribed using Maxima reverse transcriptase (Thermo Fisher Scientific, #EP0743) and oligo-dT in a total reaction volume of 20 μl of PCR buffer, adhering to the manufacturer's protocol for 60 min at 55 °C. Quantitative PCR (qPCR) was performed on an ABI 7500 system (Applied Biosystems) using Taqman gene expression assays (Thermo Fisher Scientific): SLC5A5 (Hs00950365_m1); NOX4 (Hs04980925_m1) and TSHR (Hs01053846_m1).

### Tissue samples and Immunohistochemical study

Twenty one patients with metastatic radioactive iodine refractory BRAF^V600E^-mutated differentiated thyroid cancer were included in a phase II redifferentiation trial with dabrafenib-trametininb and ^131^I. The study design, the characteristics of the patients and the trial results were published 13. Fifteen tumors from these patients were analyzed by immunohistochemistry (Supplemental Table 1). The immunohistochemical (IHC) study was performed on 3-μm-thick deparaffinized slides obtained from paraffin blocks on Ventana Automated Immunostainer (BenchMarker Ultra, Ventana Medical System, Inc,) according to the manufacturer's manual. All the technical part was handled by the experimental and translational pathology platform at Gustave Roussy (PETRA). Primary monoclonal (M) and polyclonal (P) antibodies were directed to: NOX4 (home-made antibody mAb8E9 14; 1/500), MSH2 (Millipore; clone FE11; 1/20); MSH6 (BD Bioscience; clone 44; 1/400); OGG1 (antibodies online; 6940242; 1/200); p-SMAD3 (ABCAM; ab51451; 1/500); PAX8 (Diagomics; C-P008; prediluted) and NKX2.1 (Diagomics; 8G7G3/1; prediluted). The specificity of the reactions was verified by appropriate positive and negative controls for each antibody.

IHC slides were scored by a pathologist using a standard light microscope. IHC slides with significant tissue artifacts in the tumor area that would not allow IHC assessment were excluded from analysis. All scores were performed at 20X magnification. Each markers expression scoring was assessed in malignant cells using a standard microscope and a semi-quantitative approach combining both the percentage of positive cells (0-100) and the intensity of tumor immunostaining (0, no staining; 1+, for weak, 2+, for moderate; and 3+, for strong expression), and then an H-score was calculated using the following formula: H-score = [1 × (% 1+ cells) + 2 × (% 2+ cells) + 3 × (% 3+ cells)]. Any immunoreactivity with an intensity of less than 1+ was considered background or nonspecific (“0”). The H-score range from 0 (no tumor staining) to 300 (strong staining of all tumor cells analyzed).

Statistical methods: Normality of H-score between the markers was assessed with the Shapiro-Wilk tests. Spearman's coefficient was used to assess the correlation between each H-score. Correlation was judged very strong from 1 to 0.9, strong from 0.9 to 0.7, moderate from 0.7 to 0.5, low from 0.5 to 0.3 and poor from 0.3 to 0. The alpha risk was set to 0.05.

### ChIP-qPCR assays

ChIP assays were carried out on BCPAP cells using a ChIP-IT Express kit (Active Motif, #53008), following the manufacturer's instructions. Briefly, the cross-linked cell chromatin was sheared through sonication for 15 min at 40 W (duty factor: 20%, peak incident power:200, cycles per burst: 200) with a Covaris S220 (Woodingdean, UK). Subsequently, the chromatin fragments were immunoprecipitated with 1 µg of anti-NKX2.1 (TTF1) antibody (Cell Signaling Technology, #12373, RRID: AB_2797895) or an equal amount of rabbit IgG isotype control (Agilent, #0903). The DNA bound to the chromatin immunoprecipitates was eluted and analyzed using qPCR with FastStart Universal SYBR Green Master (ROX) (Sigma/MERK, #4913850001) for detecting the TG proximal promoter sequence with specific primers (TG forward: GAGTAGACACAGGTGGAGGGA and TG reverse: GCTTTTATAGAGCTGCCGTTGG). The fold enrichment of the ChIP samples, relative to the IgG samples, was calculated via the slope of a standard curve established by performing qPCR with the primer set on known DNA quantities of input DNA (Active Motif).

### γH2AX ChIP-seq analysis

Enriched DNA from ChIP and Input DNA fragments were end-repaired, extended with an 'A' base on the 3′end, ligated with indexed paired-end adaptors (NEXTflex, Bioo Scientific) using the Bravo Platform (Agilent), size-selected after 4 cycles of PCR with AMPure XP beads (Beckman Coulter) and amplified by PCR for 10 cycles more. The final libraries were purified, pooled together in equal concentrations and subjected to paired-end sequencing (100 cycles: 2x50) on Novaseq-6000 sequencer (Illumina) at Gustave Roussy.

Quality control of reads was performed by FastQC (RRID:SCR_014583) 15, then good reads were mapped with BWA (RRID:SCR_010910) 16 to the hg38 reference from UCSC and the PCR duplicates were identified by SAMtools (RRID:SCR_002105) 17. Discared reads were those not marked as primary alignments, were unmapped, were mapped to multiple locations, and were not paired. Those evaluation and filtering were carried out by SAMtools 17. The broad peaks were called by MACS2 18 with a broad-cutoff parameter set to 0.05. Homer 19 annotation of peaks was performed to evaluate GC bias between samples, and PCA and correaltion Heatmap were made by deepTools 20. The consensus peaksets by condition were identified by BEDTools (RRID:SCR_006646) 21. Reads marked as duplicates or were mapped to blacklisted regions, were removed, then signal enrichment was scaled to 1 million mapped reads, then normalized to input sample by deepTools 20. To merge signal by condition, WiggleTools 22 was used to create a mean merged Bedgraph by condition, then convert it into BigWig file with the UCSC bedGraphToBigWig 23 function.

Next, ChIPseeker 24 R package was used to plot the consensus peaks coverage along chromosomes (under R 4.3.1). All computations used conda 25 environments, making results highly reproducible.

### ATAC sequencing analysis

For ATAC-sequencing, cells from BCPAP, 8505C, and PDX were extracted and the library preparation was conducted according to the instructions provided in the ATAC-Seq Kit (Active Motif, #53150). The quality of raw reads was assessed using FastQC 15 and FastQScreen 26, indicating good overall quality for ATACSeq. Graft reads from ATAC-Seq was mapped onto the human genome from Ensembl, version GRCh38.104. We used Sambamba 27 to remove reads with mapping quality below 30, as well as any reads without mates mapped in a proper pair. Although duplicate reads were marked with Sambamba, they were not eliminated for downstream analysis. We shifted ATAC-Seq reads using DeepTools 28, as proposed in the protocol by Buenrostro et al. 29. Additional quality controls for ATAC-Seq were performed with ATACSeqQC 30. Since the reads were previously shifted, ATAC-Seq was called using the same parameters as other libraries. We annotated peaks using Homer 19 and conducted quality controls for peak calling with DeepTools, ChipSeeker 24, and Python scripts developed in-house. The differential peak analysis was performed using edgeR 31 along with CSAW 32.

### Quantification and statistical analysis

Statistical analyses were performed using GraphPad Prism software. Data were analyzed by Student's t-test, with the minimum level of significance set at p<0.05.

## Results

### NOX4 is involved in oxidative DNA damage in BRAF-mutated thyroid cells

**Figure 1:**
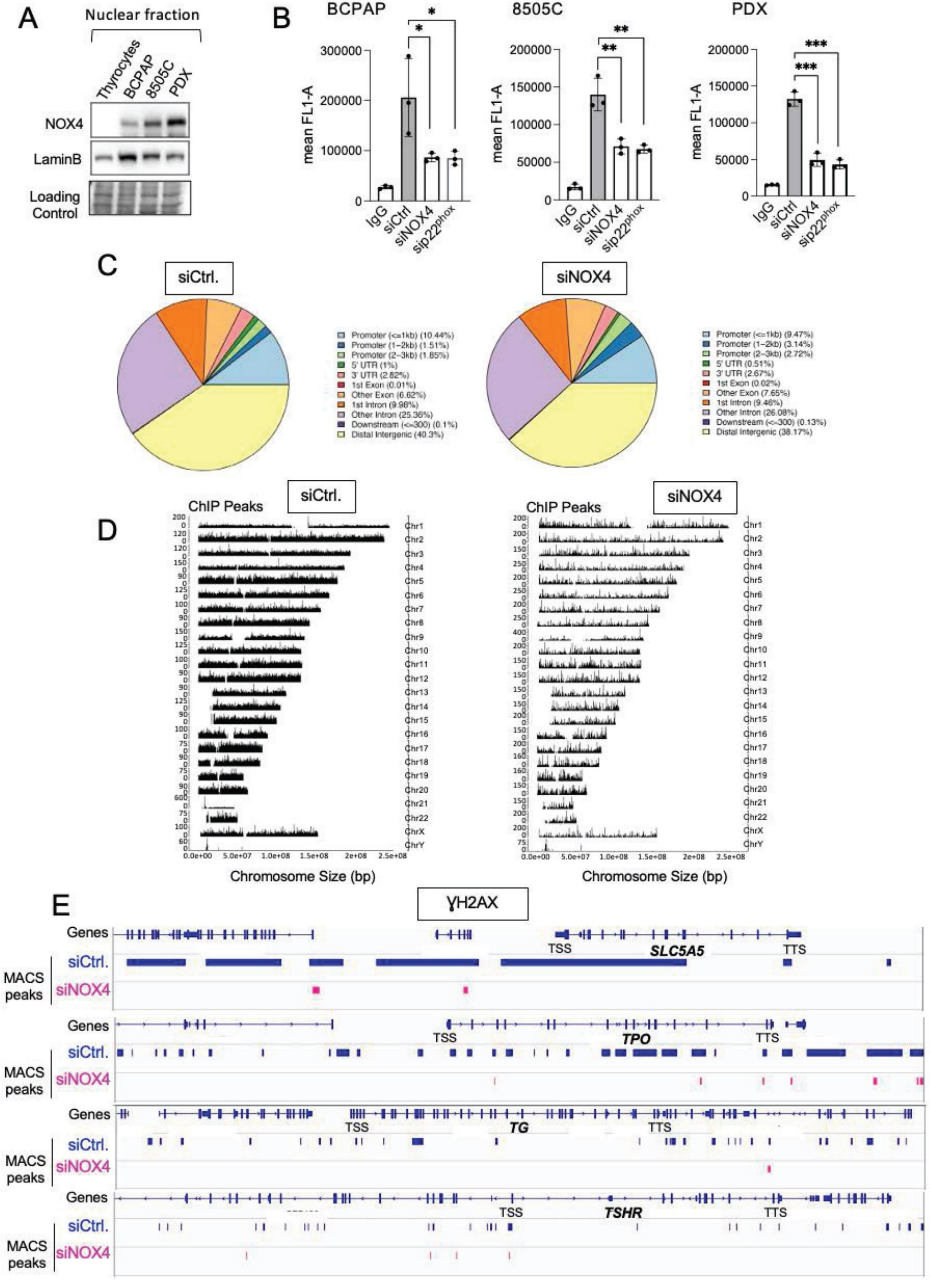
Knockdown of NOX4 or p22^phox^ reduces oxidative-DNA damage. A) Western-blot analysis of NOX4 protein expression in nuclear fractions of thyrocytes and BRAF-mutated thyroid cells (n=2) B) Quantification of 8-oxoG of BRAF-mutated thyroid cells by FACS analysis of immune-stained 8-oxoG cells. The cells were transduced with siRNA control or siRNA against NOX4 or p22^phox^ for 72 h. Cells were incubated with an anti-8oxoG or a mouse IgG2B (isotype control). Graphs show the quantification of the fluorescence mean. Values are mean ± SE. *p < 0.05, **p < 0.01, and ***p < 0.001 (n = 3). C) Genomic distribution of γH2AX in BCPAP cells depleted or not for NOX4 (72h). Two replicates for each condition (siCtrl. and siNOX4) were pooled. D) Distribution of γH2AX on genome for BCPAP cells depleted or not for NOX4. The x axis corresponds to the position of the peaks along chromosomes; the y axis corresponds to the MACS2 score (-10*(log10(q_value)). E) γH2AX enrichment peaks on selected genes in conditions siRNA control and siRNA NOX4.

NOX4 is the only NOX with constitutive ROS-generating activity directly depending on its gene expression 33. It is active in various intracellular compartments, including the nucleus. Compared to normal thyrocytes, NOX4 appears to be highly expressed in the nuclear fractions of three human cancer cell lines containing the BRAF^V600E^ mutation, with a higher expression in 8505C and the cell line established from Patient-Derived xenograft (PDX563) (Figure 1A). The membrane protein, p22^phox^, serves as its functional partner and regulates both the stability and function of NOX4 34. RNAi-mediated depletion of either NOX4 or p22^phox^ in all BRAF^V600E^-mutated thyroid cell lines significantly decreases the nuclear H_2_O_2_ level, as evaluated by FACS using NucPE1, a nuclear-localized fluorescent H_2_O_2_ sensor 12 (Figure S1A and S1B). Based on these results, we theorized that NOX4-dependent ROS production could be involved in oxidative DNA damage like the formation of 8-oxo-7,8-dihydroguanine (8-oxoG). The knockdown of either NOX4 or p22^phox^ significantly decreased the level of 8-oxoguanine as measured by cytometry in the three cell lines (Figure 1B).

The phosphorylated form of histone H2AX (γH2AX) at Ser^139^ is used as a marker of DNA damage. Next, we performed a Chromatin immuno-precipitation, followed by a high-throughput sequencing analysis of γH2AX distribution. Analysis of the genomic distribution of γH2AX peaks from biological replicates showed that they were localized within intergenic and intron regions (Figure 1C). The mapping of γH2AX reads on the genome for BCPAP cells clearly showed that γH2AX enrichment was altered by RNAi-mediated NOX4 depletion (Figure 1D). The sequence alignment analysis highlighted that γH2AX enrichment, occurring at genomic regions including the *SLC5A5* (NIS), *TPO*, *TG*, and *TSHR* genes, was impacted by this depletion (Figure 1E).

### NOX4-derived ROS mediates recruitment of MSH2, MSH6, and OGG1 to chromatin

8-oxodG is repaired either by the base excision repair (BER) pathway, a multistep process requiring several activated proteins like DNA glycosylase OGG1, or by the mismatch repair (MMR) system 35, 36. The main mismatch-binding factor in human cells is MutS, which consists of a heterodimer of MSH2 and MSH6; it has been proven to play a role in repairing clustered oxidative DNA lesions 10. From TCGA data 37, we previously compared the mRNA expression levels of MSH2 and MSH6 in BRAF^WT^-PTCs (n = 170) and BRAF^MUT^-PTCs (n = 220). Both MSH2 and MSH6 showed a significant increase in PTCs containing a BRAF mutation 38. To investigate whether MSH2 and MSH6 are regulated by mutated BRAF we transduced primary human thyrocytes with expression vector carrying cDNA for BRAF^V600E^. Western-blot analysis shows that overexpression of the activated oncogene induced an increase in both MSH2 and MSH6 protein levels in cytosolic and chromatin fractions (Figure S2). Thus BRAF^V600E^ regulates MSH2 and MSH6 protein expression levels.

**Figure 2:**
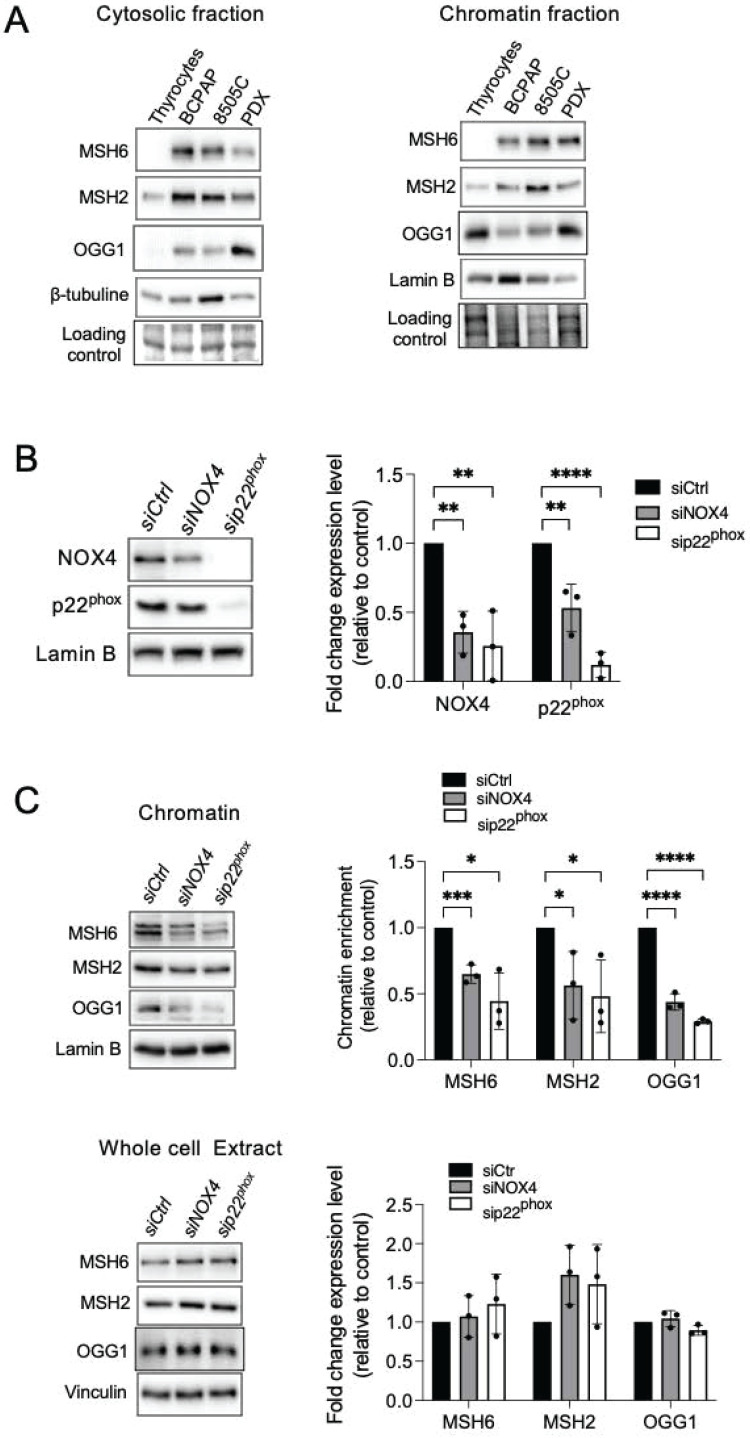
Knockdown of NOX4 or p22^phox^ reduces the recruitment of OGG1, MSH2, and MSH6 to chromatin. A) Western-blot analysis of MSH6, MSH2, and OGG1 protein expression levels in cytosolic and chromatin fractions of BRAF^V600E^-mutated thyroid cells (n=2). B) Western blot analysis of NOX4 and p22^phox^ protein expression levels in nuclear fractions 72 h after knocking down of NOX4 or p22^phox^ by RNA interference in BCPAP cells. C) Western blot analysis of MSH6, MSH2, and OGG1 protein expression levels in chromatin fractions and whole-cell extracts 72 h after knockdown of NOX4 or p22^phox^ by RNA interference in BCPAP cells. Densitometry quantification of protein levels normalized to Lamin B or Vinculin levels and presented as chromatin enrichment or fold change compared with control cells. Values are mean ± SE. *p < 0.05, ***p < 0.001 and ****p<0.0001 (n = 3).

We next compared the levels of MSH2, MSH6, and OGG1 proteins in both cytosolic and chromatin fractions of thyrocytes and BRAF-mutated thyroid cells (Figure 2A). The levels of these DNA repair proteins were elevated in the cytosolic fractions of tumor cells compared to thyrocytes, with increased levels of MSH2 and MSH6 bound to chromatin. These findings show that BRAF^V600E^ regulates the expression and the recruitment of these proteins to chromatin in thyroid cancer cells.

Given the decrease in MSH2, MSH6, and OGG1 chromatin binding upon treating BRAF^V600E^ mutated thyroid cells with N acetyl cysteine (NAC), an antioxidant, or with Diphenylene Iodonium (DPI), an inhibitor of NADPH oxidases (Figures S3A and S3B), we studied their binding to chromatin after knocking down NOX4 and p22^phox^. Interestingly, NOX4 or p22^phox^ depletion significantly decreased the amount of MSH2, MSH6, and OGG1 bound to chromatin without altering their overall levels in the three cell lines (Figures 2B and 2C, Figures S4A and S4B, Figures S5A and S5B).

### NOX4-derived ROS promotes the recruitment of DNMT1 protein to chromatin

**Figure 3:**
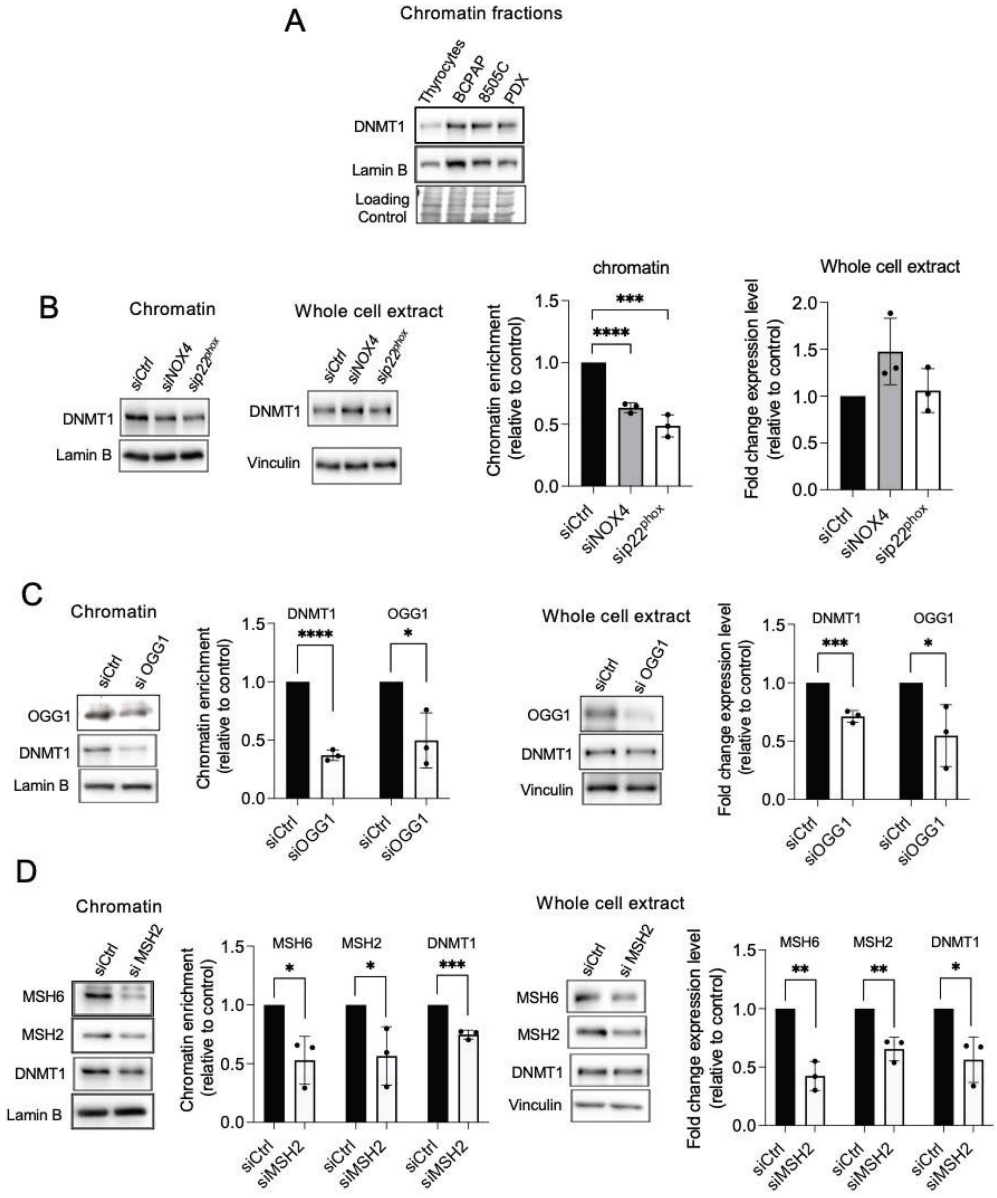
Knockdown of NOX4 or p22^phox^ as well as DNA repair proteins reduce the recruitment of DNMT1 to chromatin. A) Western-blot analysis of DNMT1 in chromatin fraction of thyrocytes and BRAF^V600E^-mutated thyroid cells (n=2). B) Western blot analysis of DNMT1 protein expression in chromatin fractions and whole-cell extracts 72 h after knocking down of NOX4 or p22^phox^ by RNA interference in BCPAP cells. C) Western blot analysis of DNMT1 protein expression in chromatin fractions and whole-cell extracts 72 h after knocking down of OGG1 by RNA interference in BCPAP cells. D) Western blot analysis of DNMT1 protein expression in chromatin fractions and whole-cell extracts 48 h after knocking down of MSH2 by RNA interference in BCPAP cells. Densitometry quantification of protein levels normalized to Lamin B or Vinculin levels and presented as chromatin enrichment or fold change compared with control cells. Values are mean ± SE. *p < 0.05, **p < 0.01, ***p < 0.001 and ****p<0.0001 (n = 3).

The DNA methylases, DNMTs, are known as major mediators of DNA methylation. BRAF^V600E^ controls the expression of DNMT1 39. Consistent with this, DNMT1 was found to be elevated in the chromatin fractions of three human cancer cell lines harboring the BRAF^V600E^ mutation (Figure 3A). Treating BRAF-mutated thyroid cells with NAC, DPI, or siRNA directed against NOX4 or p22^phox^ decreased the binding of DNMT1 to chromatin (Figure 3B, Figures S6A-S6D).

### OGG1 and MMR proteins are involved in the recruitment of DNMT1 to chromatin in BRAF-mutated thyroid cancer cells

The mismatch repair proteins MSH2 and OGG1 regulate the recruitment of DNMT1 to sites of oxidative DNA damage 8. Thus, we further evaluated if these proteins regulate DNMT1 chromatin binding in BRAF-mutated thyroid cancer cells. Depletion of OGG1 or MSH2 decreases the binding of DNMT1 to the chromatin as well as its expression in whole-cell extracts (Figure 3C and 3D). These findings suggest that these DNA repair proteins play a role in DNMT1 recruitment to areas of oxidative DNA damage produced by NOX4.

**Figure 4:**
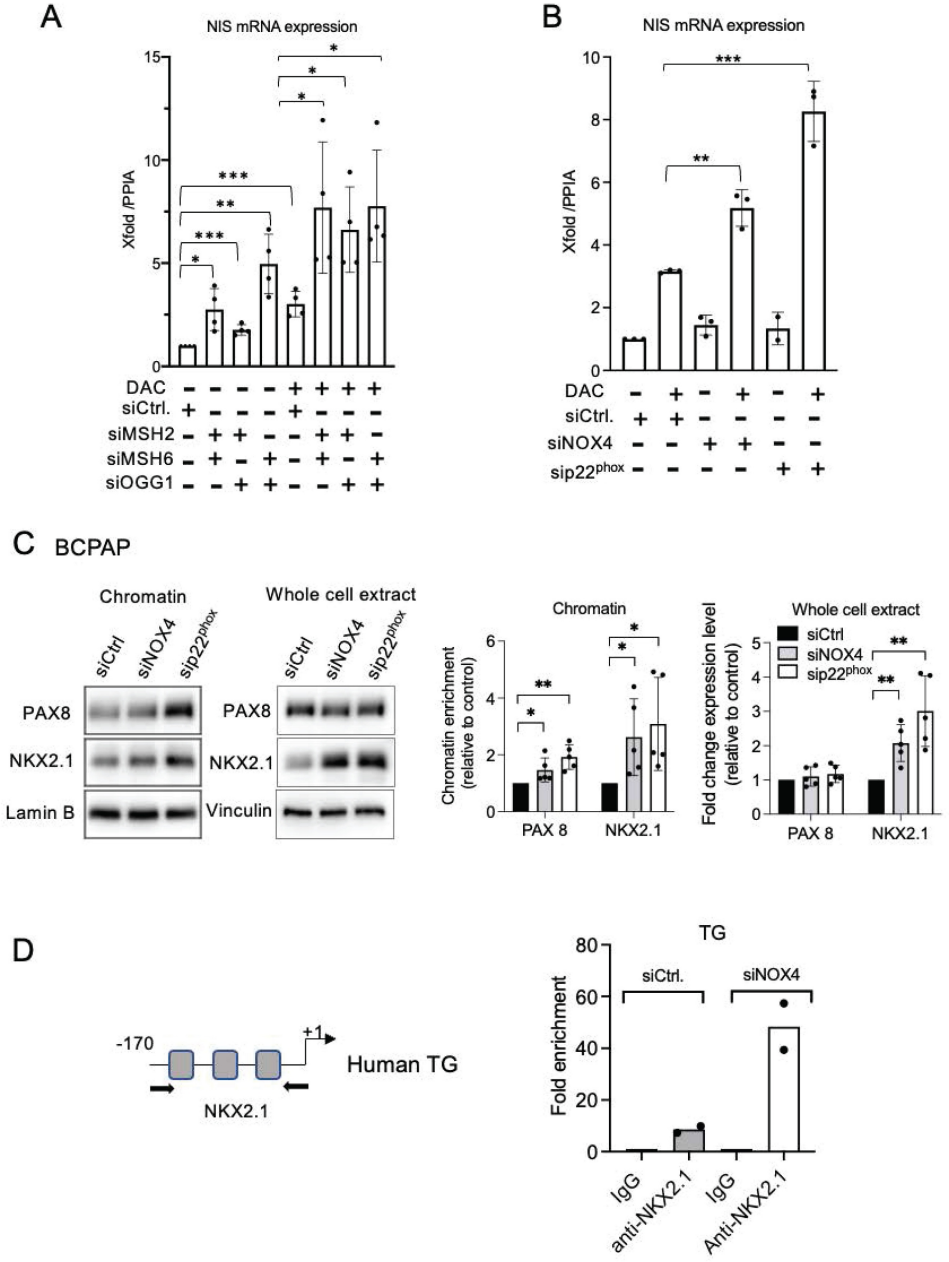
DNMT inhibition and knockdown of MSH2, MSH6, and OGG1 synergize to increase NIS mRNA expression. A) qRT-PCR analysis of NIS mRNA levels in BCPAP cells transfected with siRNA control or/and siRNA MSH2 or/and siRNA MSH6 or/and siRNA OGG1 and 24 h later treated for an additional 48 h in the presence or the absence of 1 µM DAC. B) qRT-PCR analysis of NIS mRNA levels in BCPAP cells transfected with siRNA control or siRNA NOX4 or siRNA p22^phox^ and 24 h later, treated for an additional 48 h in the presence or the absence of 1 µM DAC. Values are mean ± SE. *p < 0.05, **p < 0.01, and ***p < 0.001. C) Western blot analysis of PAX8 and NKX2.1 protein expressions in chromatin fraction and whole-cell extract 72 h after knocking down of NOX4 or p22^phox^ by RNA interference in BCPAP cells. Densitometry quantification of protein levels normalized to Lamin B or Vinculin levels and presented as chromatin enrichment or fold change compared with control cells. Values are mean ± SE. *p < 0.05 and **p < 0.01 (n = 5). D) ChIP-qPCR assays performed with BCPAP cells transfected with siRNA control and with BCPAP cells transfected with siRNA NOX4 immunoprecipitated with control IgG or anti-NKX2.1 antibody and analyzed by qPCR at TG promoter (two independent replicates).

Combining siRNA-mediated MSH2/MSH6 or OGG1 knockdown with Decitabine (DAC), a DNMT inhibitor, resulted in a significant increase in the reactivation of the *SLC5A5* gene (Figure 4A, Figure S7A). The effects of single siRNAs were also evaluated but these induced a less pronounced increased (Figure S7B). This indicates that MMR and OGG1 proteins cooperate with DNMT1 to maintain the silencing of this gene. We were able to reproduce these results by combining siRNA-mediated NOX4 or p22^phox^ depletion with DAC (Figure 4B). Thus, NOX4 mediates the recruitment of MMR, OGG1, and DNMT1 proteins on damaged chromatin and these may work synergistically to repress genes such as *SLC5A5*.

### NOX4-derived ROS prevents the recruitment of transcription factors NKX2.1 and PAX8

The promoters of the primary thyroid differentiation markers are regulated by the specific combination of the transcription factors NKX2.1 and PAX8 2. NAC or DPI treatment of BRAF-mutated thyroid cells increased the binding of PAX8 and NKX2.1 to chromatin, as well as their total level of expression, indicating that the recruitment and expression of these proteins are redox-sensitive (Figures S8A and S8B). Importantly, the knockdown of NOX4 or p22^phox^ significantly increased the affinity of both transcription factors for chromatin in the analyzed BRAF-mutated thyroid cell lines BCPAP and PDX (Figure 4C, Figure S8C and Figure S9A). Since PAX8 was undetectable in 8505C cells, only the effect on NKX2.1 was analyzed, and the same result was obtained (Figure S9B). Three sites on the TG proximal promoter are recognized by NKX2.1 40. Using a ChIP qPCR assay, we observed that the knockdown of NOX4 increased NKX2.1 binding to the promoter (Figure 4D).

**Figure 5:**
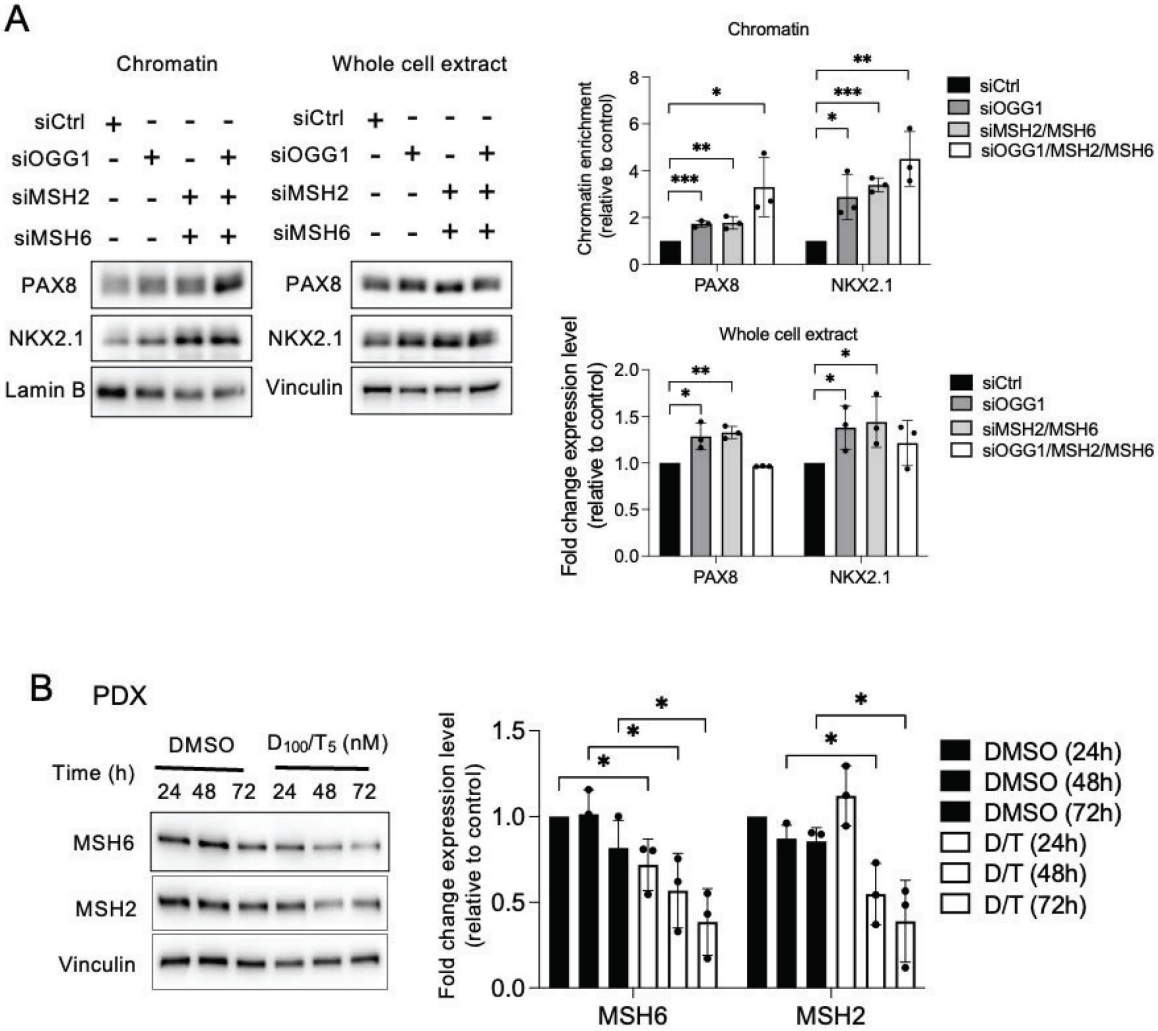
OGG1, MSH2, and MSH6 inhibit recruitment of PAX8 and NKX2.1 to chromatin and the MAPK pathway regulates MSH2 and MSH6 expressions. A) Western blot analysis of PAX8 and NKX2.1 protein expressions in chromatin fraction and whole-cell extract 72 h after knocking down of OGG1 (siRNA OGG1#1), MSH2, or/and MSH6 by RNA interference in BCPAP cells. Densitometry quantification of protein levels normalized to Lamin B or Vinculin levels and presented as chromatin enrichment and fold change compared with control cells. Values are mean ± SE. *p < 0.05, **p < 0.01, and ***p < 0.001 (n = 3). B) Immunoblot detection of MSH2 and MSH6 in PDX cells after treatment by the combination of dabrafenib (100 nM) plus trametinib (5 nM). Densitometric quantification of protein levels normalized to vinculin levels and presented as fold change compared with vehicle-treated cells. Student t-test is realized by comparing combination versus DMSO for each corresponding time of kinetic. Values are mean ± SE. *p < 0.05 and **p < 0.01 (n = 3).

Also, the knockdown of OGG1 or both MSH2 and MSH6 facilitated the recruitment of the two transcription factors to the chromatin in the BRAF-mutated cell lines (Figure 5A, Figures S10A-S10C). Depending on the cell line, their total level of expression also appeared to be modulated (Figures S11A-S11B, Figures S12A-S12C). These results suggest that NOX4-derived ROS prevent PAX8 and NKX2.1 from binding to the chromatin through the recruitment of DNA repair proteins to the chromatin, induced by oxidative damage.

To further investigate the influence of NADPH oxidase on genome-wide chromatin accessibility, we performed an Assay for Transposase-Accessible Chromatin using sequencing (ATAC-seq) on BCPAP, 8505C, and PDX563 tumor thyroid cells, with or without p22^phox^ depletion (Figures S13A-S13E and Figures S14A-S14F). We detected a relatively distinct footprint of TF occupancy near the aggregated PAX8 and NKX2.1 full sites in accessible chromatin regions in all control and p22^phox^-depleted cells. Moreover, the compaction level of PAX8 and NKX2.1 genes was not altered by p22^phox^ depletion. Intergenic and intron regions were the most enriched genomic features of the accessible regions in the BRAF-mutated thyroid cell lines. Most of the peaks were 0-1 kb and 10-100 kb away from TSS, a distribution not affected by p22^phox^ depletion. Lastly, the abrogation of p22^phox^ expression did not modulate accessibility to the set of thyroid differentiation marker genes.

### MSH2 and MSH6 expressions are inhibited by the combination of dabrafenib/trametinib

Clinical evidence has shown a link between MAPK pathway inhibition and increased molecular differentiation in patients, supporting that MAPK is a critical regulator of thyroid tumor differentiation 1. The combination of dabrafenib/trametinib inhibits both mutated BRAF and MEK in all cell lines, demonstrated by the inhibition of phospho-MEK and phospho-ERK (Figure S15A). A 72-h treatment of BRAF-mutated thyroid cell lines with this combination led to a downregulation of MMR proteins, indicating that the MAPK pathway controls the expression of these proteins in tumor cells (Figure 5B and Figures S15B and S15C).

### NOX4 cooperates with the MAPK pathway in the thyroid dedifferentiation process

**Figure 6:**
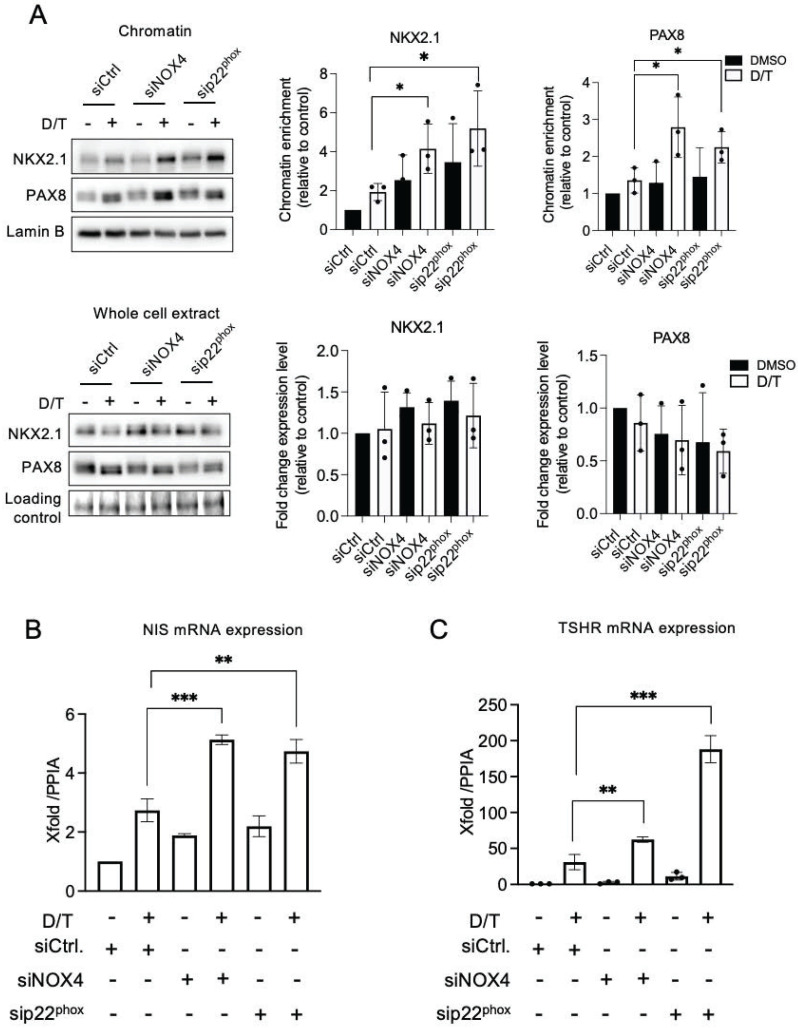
MAPK pathway inhibition and knockdown of NOX4 and p22^phox^ synergize to promote PAX8 and NKX2.1 recruitment to chromatin. A) Western-blot analysis of PAX8 and NKX2.1 protein expressions in chromatin fractions and whole-cell extracts of BCPAP cells transduced with siRNA control or siRNA NOX4 or siRNA p22^phox^ and treated with dabrafenib (100 nM) plus trametinib (25 nM) combination for 48 h. Densitometric quantification of protein levels normalized to Lamin B or loading control (red ponceau staining) and presented as chromatin enrichment and fold change compared with siRNA control-transduced cells. B) qRT-PCR analysis of NIS mRNA levels in BCPAP cells transfected with siRNA control or siRNA NOX4 or siRNA p22^phox^ and 24 h later, treated in the presence or the absence of dabrafenib plus trametinib combination for additional 48 h. C) qRT-PCR analysis of TSHR mRNA levels in BCPAP cells transfected with siRNA control or siRNA NOX4 or siRNA p22^phox^ and 24 h later, treated in the presence or the absence of dabrafenib plus trametinib combination for additional 48 h. Values are mean ± SE. *p < 0.05, **p < 0.01, and ***p < 0.001 (n = 3).

The combined knockdown of NOX4 or p22^phox^, in conjunction with dabrafenib/trametinib treatment, led to increased recruitment of NKX2.1 and PAX8 to the chromatin (Figure 6A and Figures S16A-S16B). This was associated with an increase in the expression of NIS and TSHR at the mRNA levels, which indicates that the NADPH oxidase NOX4 cooperates with the MAPK pathway in the thyroid dedifferentiation process (Figures 6B and 6C).

**Figure 7:**
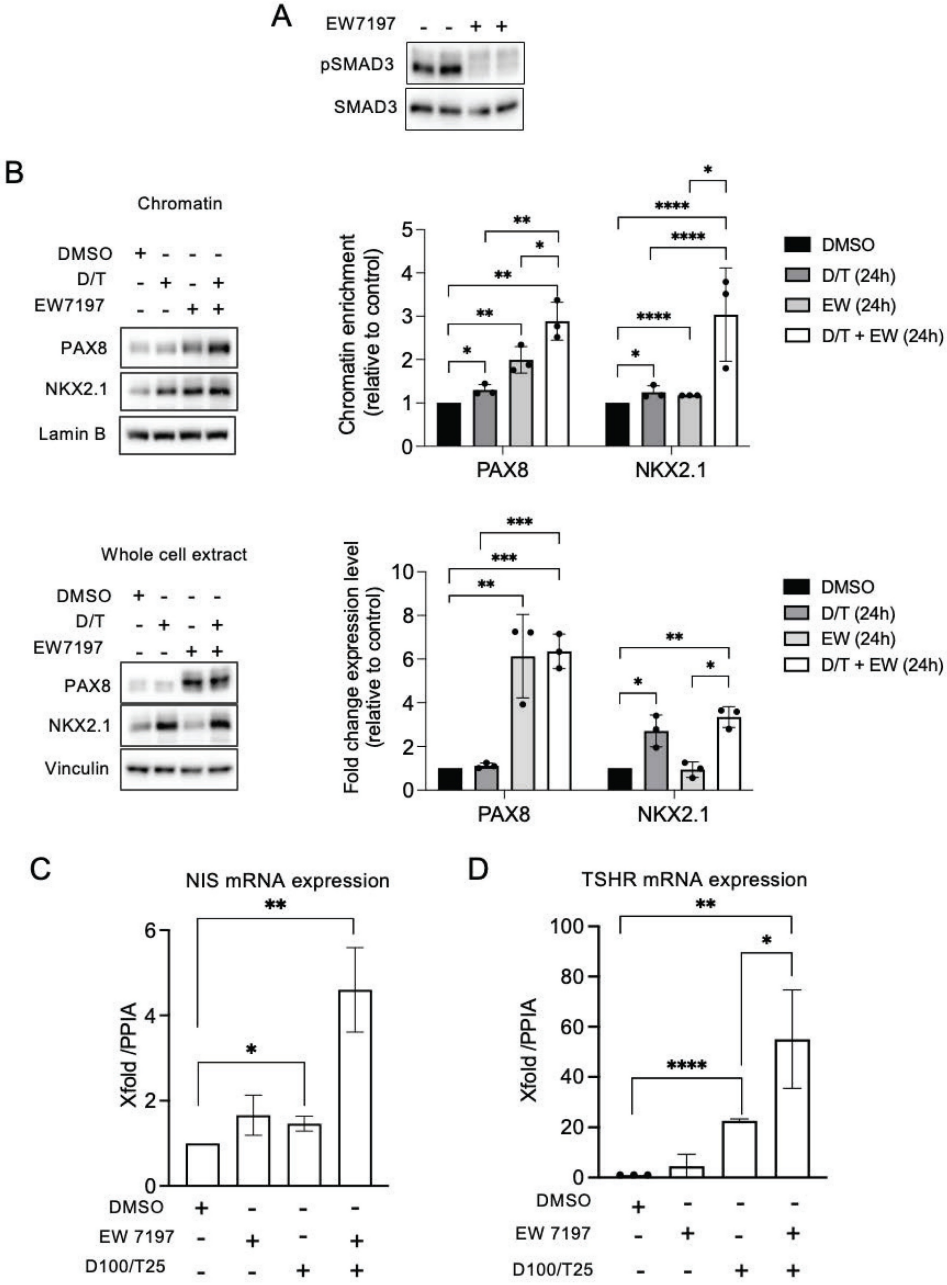
Co-inhibition of SMAD and MAPK signalling promotes PAX8 and NKX2.1 recruitment to chromatin. A) BRAF-mutated thyroid cells were treated with TGF-beta receptor inhibitor (EW7197, 1 µM) for 4 h and analysed by Western-blot for expression of pSMAD3 and SMAD3. B) Western-blot analysis of PAX8 and NKX2.1 protein expressions in chromatin fractions and whole-cell extracts of BCPAP cells treated or not with dabrafenib (100 nM) plus trametinib (25 nM) combination in the presence or the absence of TGF-beta inhibitor (EW7197, 1 µM) for 48 h. Densitometric quantification of protein levels normalized to loading control and presented as chromatin enrichment or fold change compared with vehicle-treated cells. C and D) qRT-PCR analysis of NIS and TSHR mRNA levels in BCPAP cells treated or not with dabrafenib plus trametinib combination in the presence or the absence of TGF-beta inhibitor (EW7197) for 48 h. Densitometry quantification of protein levels normalized to Lamin B or Vinculin levels and presented as fold change compared with control cells. Values are mean ± SE. *p < 0.05, **p < 0.01, ***p < 0.001 and ****p<0.0001 (n = 3).

We have previously shown that the TGF-β1 signaling pathway upregulates NOX4 in BRAF-mutated thyroid cells at the transcriptional level in a Smad3-dependent manner 7. Luckett et al. used a genetically engineered mouse model of BRAF mutant thyroid cancer to show that suppressing both the MAPK and pSMAD pathways led to enhanced radioiodine uptake in mouse cancer cells 41. Considering these results, we evaluated the combined effect of Dabrafenib/Trametinib and Vactosertib (EW7197), an inhibitor of the TGF-β1 pathway, on human BRAF mutated thyroid cancer cells. EW7197 increased the recruitment of PAX8 and NKX2.1 to the chromatin, and this effect was potentiated when the inhibitor was combined with MAPK kinase pathway inhibitors. This led to an increase in both TSHR and NIS mRNA expressions (Figures 7A-7C, Figures S17A and S17B).

### NOX4, OGG1, MSH2, MSH6 and phospho-Smad3 are upregulated in Radioactive Iodine Refractory (RAIR) BRAF^V600E^-mutated thyroid cancer

**Figure 8:**
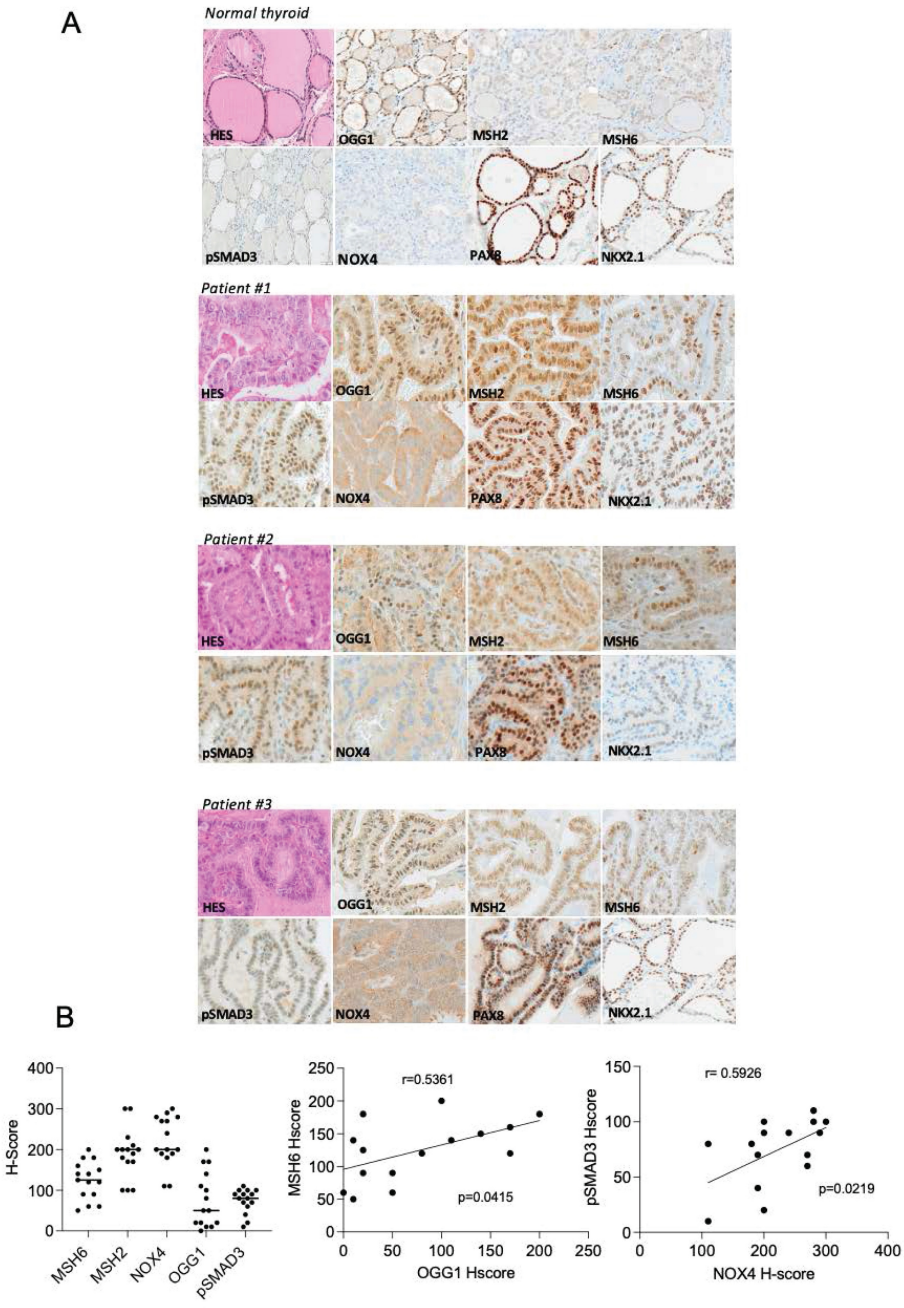
NOX4, oxidative DNA repair proteins and phospho-Smad3 are upregulated in radioactive iodine refractory BRAF^V600E^-mutated thyroid cancer. A) Immunoexpression of the different markers in normal thyroid tissue and in BRAF-mutated thyroid tumors. B) Immunohistochemical scoring system (H-Score) values determined from RAIR thyroid tumors for MSH6, MSH2, NOX4, OGG1 and phospho-SMAD3. Correlation between immunohistochemical markers.

A phase II redifferentiation trial with dabrafenib/trametinib and ^131^I in metastatic radioactive iodine refractory BRAF^V600E^-mutated differentiated thyroid cancer was previously conducted to explore therapeutic efficacy of drug combination 13. The patients (n=21) underwent 6-weeks of treatment with dabrafenib and trametinib followed by a therapeutic activity of RAI (5.5 GBq of ^131^I) administered following two rhTSH injections. Some patients displayed primary resistance to this redifferentiation strategy and one patient achieved a complete response. From 15 of primary tumor tissue of these patients, which were papillary thyroid cancers, characterized by the loss of follicular structure and the appearance of a characteristic papillary structure, we undertook to analyze NOX4, DNA repair proteins (OGG1, MSH2 and MSH6) and phospho-Smad3 expressions by immunohistochemistry (Table S1). Compared to normal tissue we identified an increased expression of NOX4, OGG1, MMR proteins and phospho-Smad3 in RAIR BRAF^V600E^ mutated tumors (Figures 8A and 8B). In contrast the two transcription factors PAX8 and NKX2.1 showed the same level of expression. We identified a significant positive correlation between MSH6 and OGG1 expression (p=0.0415, Spearman r=0.5361) and between phospho-Smad3 and NOX4 (p=0.0219, Spearman r=0.5926).

## Discussion

The findings reported here provide new insights into our understanding of the epigenetic role of 8-oxoG in BRAF^V600E^-mutated thyroid cancers, emphasizing the contribution of oxidative DNA damage in redox-mediated control of gene expression in thyroid dedifferentiation processes by altering the recruitment of two key transcription factors, PAX8 and NKX2.1.

Phosphorylation of H2AX plays a key role in DNA Damage Response (DDR) and is required for the assembly of DNA repair proteins at sites containing DSBs 42. From Genome-wide mapping of 8 oxoG and γH2AX it has been shown that there was a co-occurrence of high levels of DNA oxidation and γH2AX within a significant fraction of coding and non-coding genes in both human and mouse cells analyzed 43. The authors proposed that slower repair of 8-oxodG sites, with formation of single strand breaks (SSB) as a consequence of OGG1 and BER enzymatic activities, respectively, might lead to DSB formation within genomic regions containing either closely opposed 8-oxodG sites or individual 8-oxodG sites occurring in proximity to other kinds of lesion. In addition, isolated SSBs formed during the processing of 8-oxodG can also be converted in DSBs during S-phase.

8-oxoG DNA damage, in conjunction with OGG1, can influence transcriptional regulation (either activation or repression) by manipulating transcription factor homing or the recruitment of chromatin remodelers 44. A significantly higher expression of the OGG1 gene and its corresponding protein has recently been noted in lesions of papillary thyroid cancer (PTC) compared to benign lesions 45. OGG1 is drawn to open chromatin regions where 8-oxoG effectively recruits BER enzymes 46. Pro-oxidant conditions can also lead to catalytic inactivation of OGG1 via cysteine oxidation. However, this does not interfere with damage-specific binding. Some studies have now described the non-catalytic functions of OGG1 as a transcriptional modulator that either interfere with transcription factor binding or facilitate DNA binding and transcription modulation 47. OGG1 cannot excise 8-oxoG within clustered lesions. Alternate DNA repair systems such as the non-canonical Mismatch Repair (MMR) system, including proteins MSH2 and MSH6, manage 8-oxoG in these situations. This is particularly pertinent in the repair of clustered oxidative damage found in GC-rich regions of the genome, including promoter CpG islands 10. Higher levels of MSH2 have been identified in malignant thyroid tumors in comparison to benign tumors 48. In the present work we show that both OGG1 and MMR proteins are up-regulated in iodine-refractory BRAF^V600E^-mutant metastatic differentiated thyroid cancer, indicating a functional activation of these genes. Thyroid tumors exhibit a low overall density of somatic mutations compared to other tumors, suggesting efficient support from both the BER and MMR systems. Our findings reveal that NOX4-derived 8-oxoG can be converted into transcription-blocking damage by OGG1 and MMR proteins in BRAF^V600E^-mutated thyroid cancer cells.

NKX2.1 (or TTF1) and PAX8 are integral to thyroid organogenesis and the continued differentiation of thyrocytes 2. Previous research revealed a redox regulation of Pax8 and NKX2.1 is implicated in their heightened DNA-binding activities prompted by thyrotropin in rat thyroid FRTL-5 cells 49. ATAC seq data analysis shows that the alteration of NOX4 activity does not lead to a change in chromatin accessibility for the two transcription factors. These findings indicate that the discordance between chromatin accessibility and transcriptional activity arises from 8 oxoG DNA lesions, which, by facilitating the engagement of OGG1 and MMR proteins with damaged chromatin, impacts the binding of these two transcription factors to chromatin in BRAF-mutated thyroid cancer cells. Thus, this contributes to the process of thyroid dedifferentiation. Interestingly, PAX8 and NKX2.1 are expressed in the radioactive iodine refractory BRAF^V600E^-mutated thyroid tumours analyzed, highlighting that a decrease in their binding to chromatin and not in their level of expression may be one of the causes of the refractoriness of these tumors.

Loss of differentiation features correlates with the degree of MAPK activation, which is higher in tumors with the BRAF^V600E^ mutation. Here we demonstrate that the oncogene controls the expression of MSH2 and MSH6 through an ERK-dependent pathway, providing an additional element to the understanding of the efficacy of inhibitors of the MAPK-signaling pathway in clinic. However, the inhibition of MAPK transcriptional output did not result in sufficient redifferentiation in all treated patients. This suggests that more potent pathway inhibition may be required, or that biological factors beyond MAPK inhibition could be critical. We identified NOX4 as an additional effector working in tandem with the RAF/MEK/ERK cascade to fully repress the functions of NKX2.1 (TTF1) and PAX8.

A TGF-β1 transcriptional output signature was identified in advanced RAI-refractory human BRAF-mutant thyroid cancers 41. TGF-β1 was first demonstrated to play a significant role as a local thyroid modulator by inhibiting both growth and differentiation in various species 50. The expression of BRAF^V600E^ triggers the production of functional TGF-β1, leading to a TGF-β-driven autocrine loop facilitating the effects of the BRAF^V600E^ oncoprotein. This includes the decreased expression of NIS 51 and the promotion of cell migration, invasiveness, and EMT 52. We previously labeled NOX4 as a novel key effector of TGF-β1 in BRAF^V600E^-induced thyroid tumors 7. The pharmacologic inhibition of the TGF-beta pathway, along with the MAPK pathway, parallels the results obtained from combining NOX4 or p22^phox^ knockdown with MAPK pathway inhibition. Until now the precise mechanism underlying the additive effect of SMAD and MEK inhibition on iodide uptake remained unclear. We demonstrate that TGF-β1-induced signaling through SMAD may especially contribute to the suppression of NIS by altering the recruitment of PAX8 and NKX2.1 *via* NOX4-dependent oxidative damage. Further exploration of the translational implications of these findings in clinical trials will employ a combination of drugs targeting the MAPK pathway and the TGF-β1 pathway.

In conclusion, our findings reveal that NADPH oxidase NOX4, which has notable expression in BRAF^V600E^-mutated thyroid tumor cells and produces H_2_O_2_ in the nuclear environment, repeatedly generates oxidative DNA damage. This promotes increased retention of epigenetic modifiers, such as DNMT1, at the sites of DNA damage alongside DNA repair proteins including OGG1 and MSH2/MSH6. All these factors contribute to blocking the recruitment of two vital transcriptional factors: PAX8 and NKX2.1, which are involved in the transcription of genes associated with thyroid differentiation. The co-inhibition of the MAPK pathway by combining Dabrafenib/Trametinib (which diminishes the expression levels of MSH2/MSH6 and DNMT1) with the TGF-β1 pathway by EW7197 (which reduces NOX4 levels), enhances the recruitment of the two transcription factors to the chromatin, promoting, in particular, the re-expression of SLC5A5 encoding NIS, which is a prerequisite for radioiodine therapy in thyroid cancer.

## Supplementary Material

Supplementary figures.
