# Supplementary material for: NOX4-derived oxidative DNA damage impairs thyroid differentiation through an epigenetic mechanism in BRAF-mutated radioactive iodine refractory papillary thyroid cancer cells
Source: Int J Biol Sci. 2026 Jan 15;22(4):1674–92. doi: 10.7150/ijbs.123980 (PMC12905579; doi:10.7150/ijbs.123980)
Supplement: Supplementary file 1 — Supplementary figures. [file ijbsv22p1674s1.pdf]

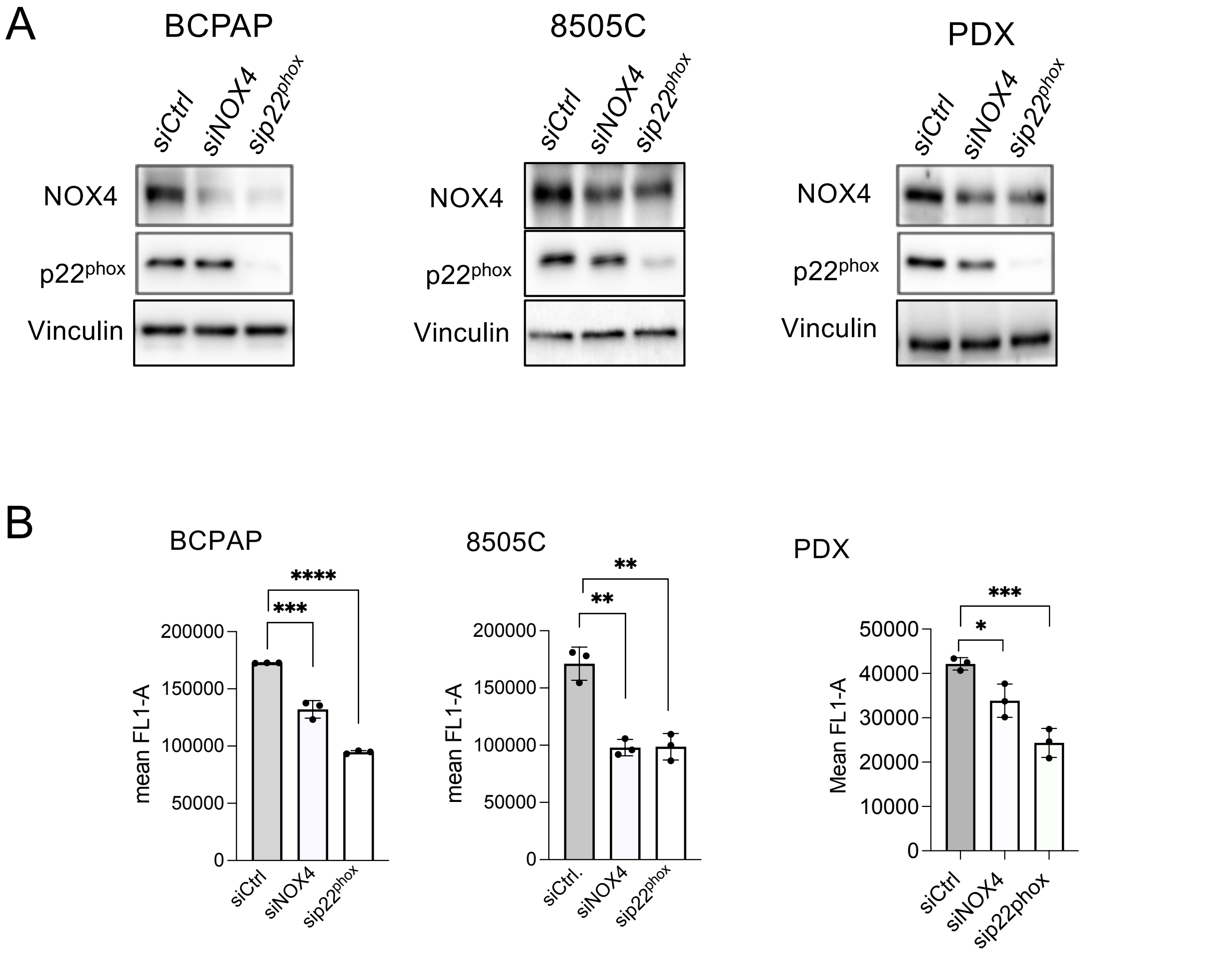

Figure S1: Knockdown of NOX4 or p22^{phox} reduces the nuclear level of H_2O_2 A) Western-blot analysis of NOX4 and p22phox protein expression levels in whole-cell extract after knocking down of NOX4 or p22phox by RNA interference in BRAF-mutated thyroid cells. B)The nuclear level of ROS was measured in BRAF-mutated thyroid cells by NucPEI fluorescence using flow cytometry. The cells were transduced with siRNA control or siRNA against NOX4 or p22^{phox} for 72 h. Graphs show the quantification of the fluorescence mean. Values are mean ± SE. *$p < 0.05$, **$p < 0.01$, ***$p < 0.001$ and ****$p<0.0001$ (n = 3).

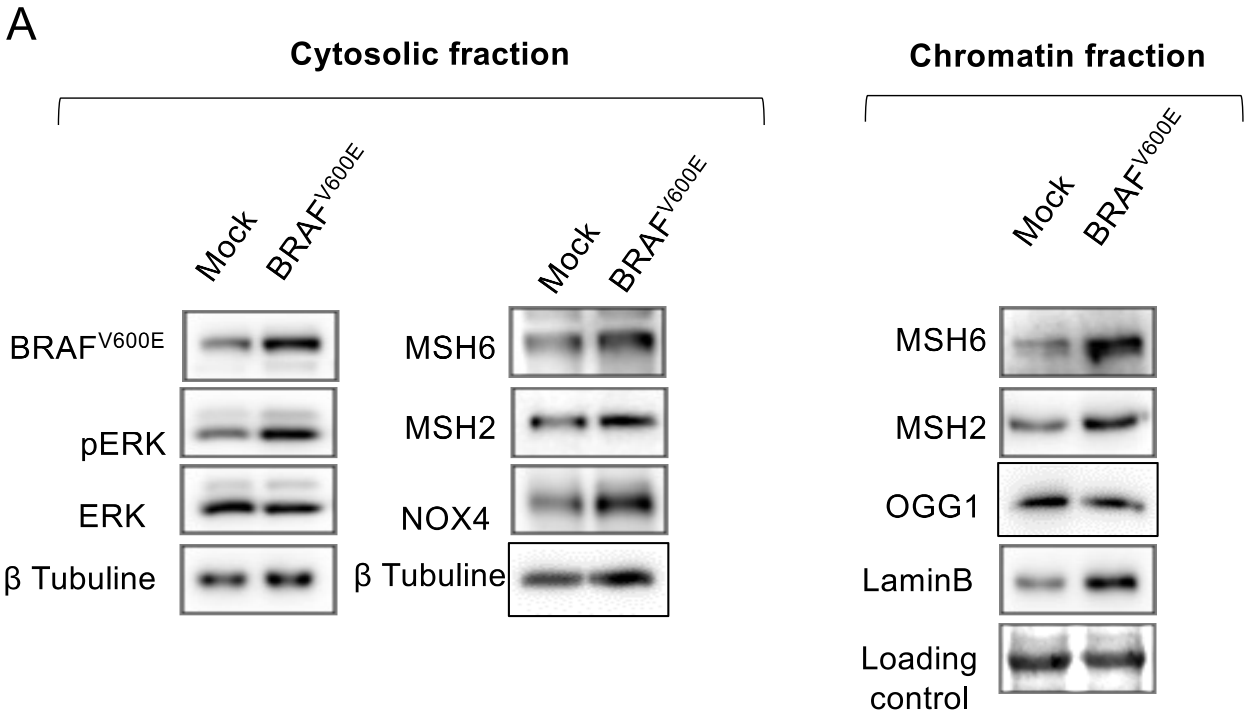

Figure S2: BRAF^{V600E} up-regulates MSH2 and MSH6 protein expression levels. a) Human primary thyrocytes were transiently transfected with empty vector (Mock) or BRAFV600E plasmids for 48h and phospho-ERK, ERK, MSH2, MSH6, OGG1 and NOX4 protein expression levels were analyzed in cytosolic and chromatin fractions by western-blot (n=2).

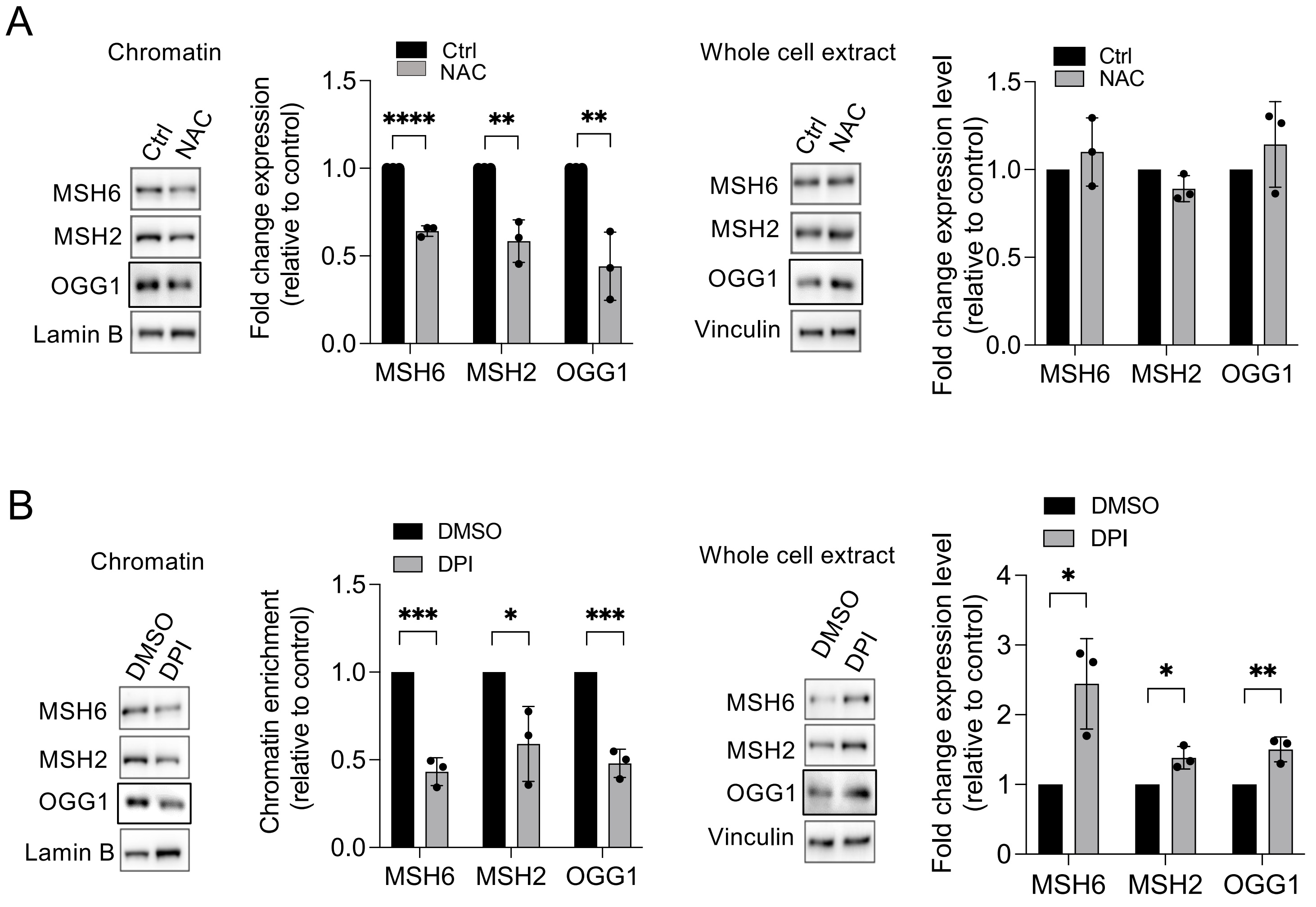

Figure S3: Anti-oxidant (NAC) or NADPH oxidase inhibitor (DPI) alters the recruitment of OGG1, MSH2, and MSH6 to chromatin in BRAF-mutated thyroid cells. A) BCPAP cells were pre-treated with 5 mM NAC for 2 h before being analysed for OGG1, MSH2, and MSH6 protein expression levels in chromatin fractions and whole-cell extract. B) BCPAP cells were pre-treated with 1 µM DPI for 6 h before being analysed for OGG1, MSH2, and MSH6 protein expression levels in chromatin fractions and whole-cell extracts. Densitometry quantification of protein levels normalized to Lamin B or Vinculin levels and presented as chromatin enrichment or fold change compared with control cells. Values are mean ± SE. *$p < 0.05$, **$p < 0.01$, and ***$p < 0.001$ ($n = 3$).

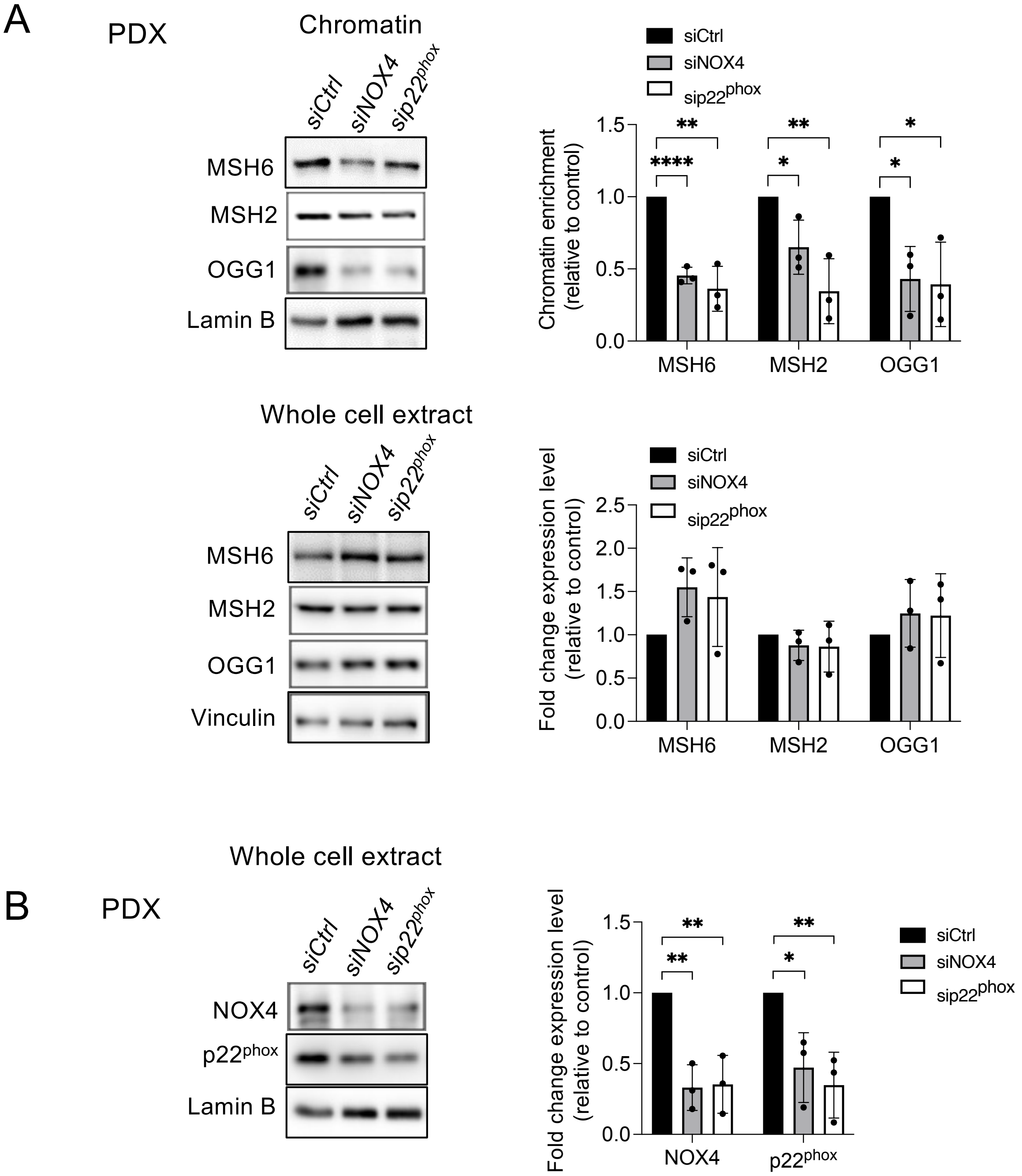

Figure S4 : Knockdown of NOX4 or p22^{phox} reduces the recruitment of OGG1, MSH2, and MSH6 to chromatin in BRAF-mutated thyroid cells. A) Western blot analysis of MSH6, MSH2, and OGG1 protein expression levels in chromatin fractions and whole-cell extracts 72 h after knocking down of NOX4 or p22^{phox} by RNA interference in PDX cells. B) Western blot analysis of NOX4 and p22^{phox} protein expression levels in whole-cell extract 72 h after knocking down of NOX4 or p22^{phox} by RNA interference in PDX cells. Densitometric quantification of protein levels normalized to loading control levels and presented as chromatin enrichment or fold change compared with siRNA control-transduced cells. Values are mean ± SE. $*p < 0.05$, $**p < 0.01$, $***p < 0.001$ and $****p<0.0001$ ($n = 3$).

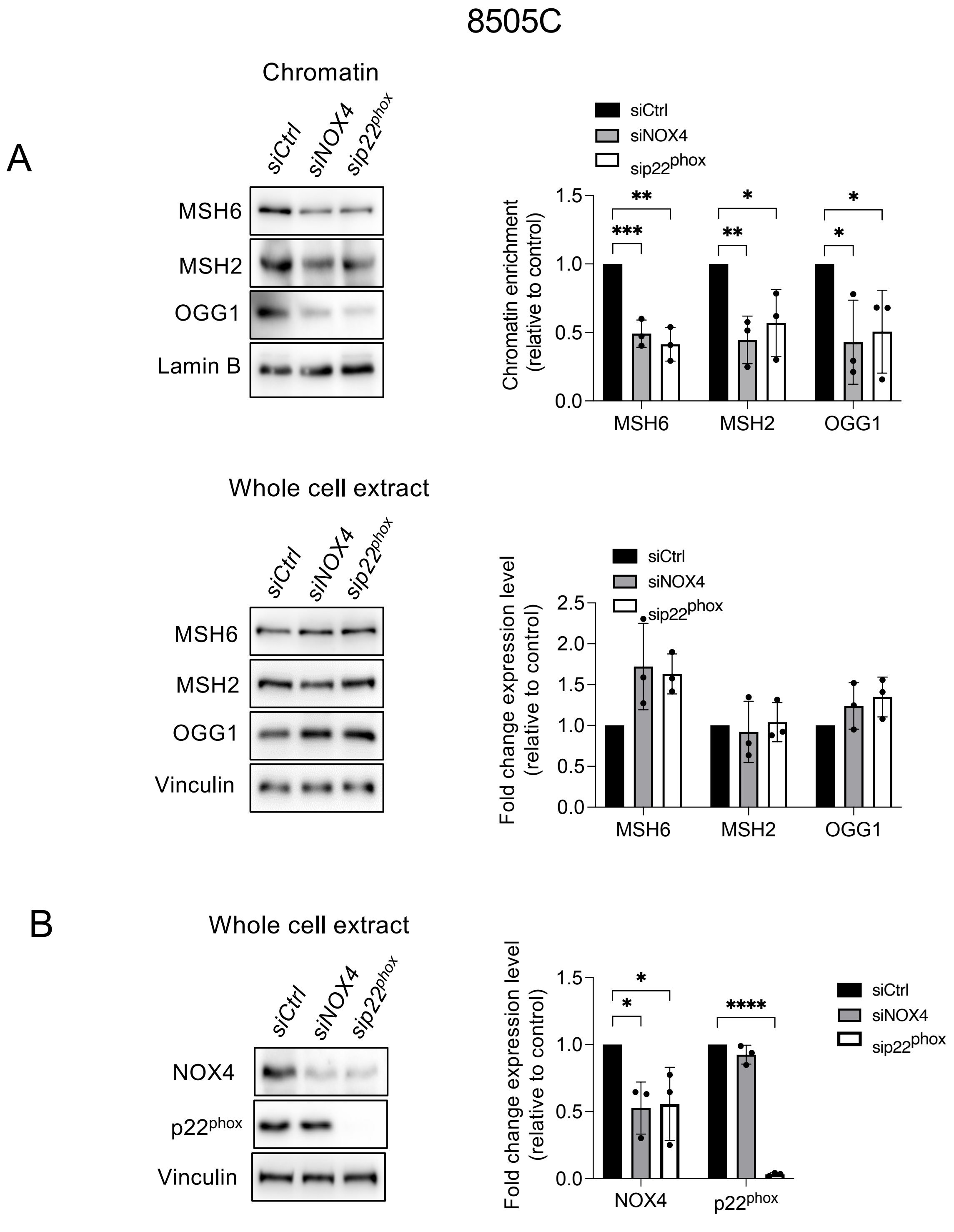

Figure S5: Knockdown of NOX4 or $p22^{phox}$ reduces the recruitment of OGG1, MSH2, and MSH6 to chromatin in BRAF-mutated thyroid cells. A) Western blot analysis of MSH6, MSH2, and OGG1 protein expression levels in chromatin fractions and whole-cell extracts 72 h after knocking down of NOX4 or $p22^{phox}$ by RNA interference in 8505C cells. B) Western blot analysis of NOX4 and $p22^{phox}$ protein expression levels in whole-cell extracts 72 h after knocking down of NOX4 or $p22^{phox}$ by RNA interference in 8505C cells. Densitometric quantification of protein levels normalized to loading control levels and presented as chromatin enrichment or fold change compared with siRNA control-transduced cells. Values are mean ± SE. *$p < 0.05$, **$p < 0.01$, ***$p < 0.001$ and ****$p<0.0001$ ($n = 3$).

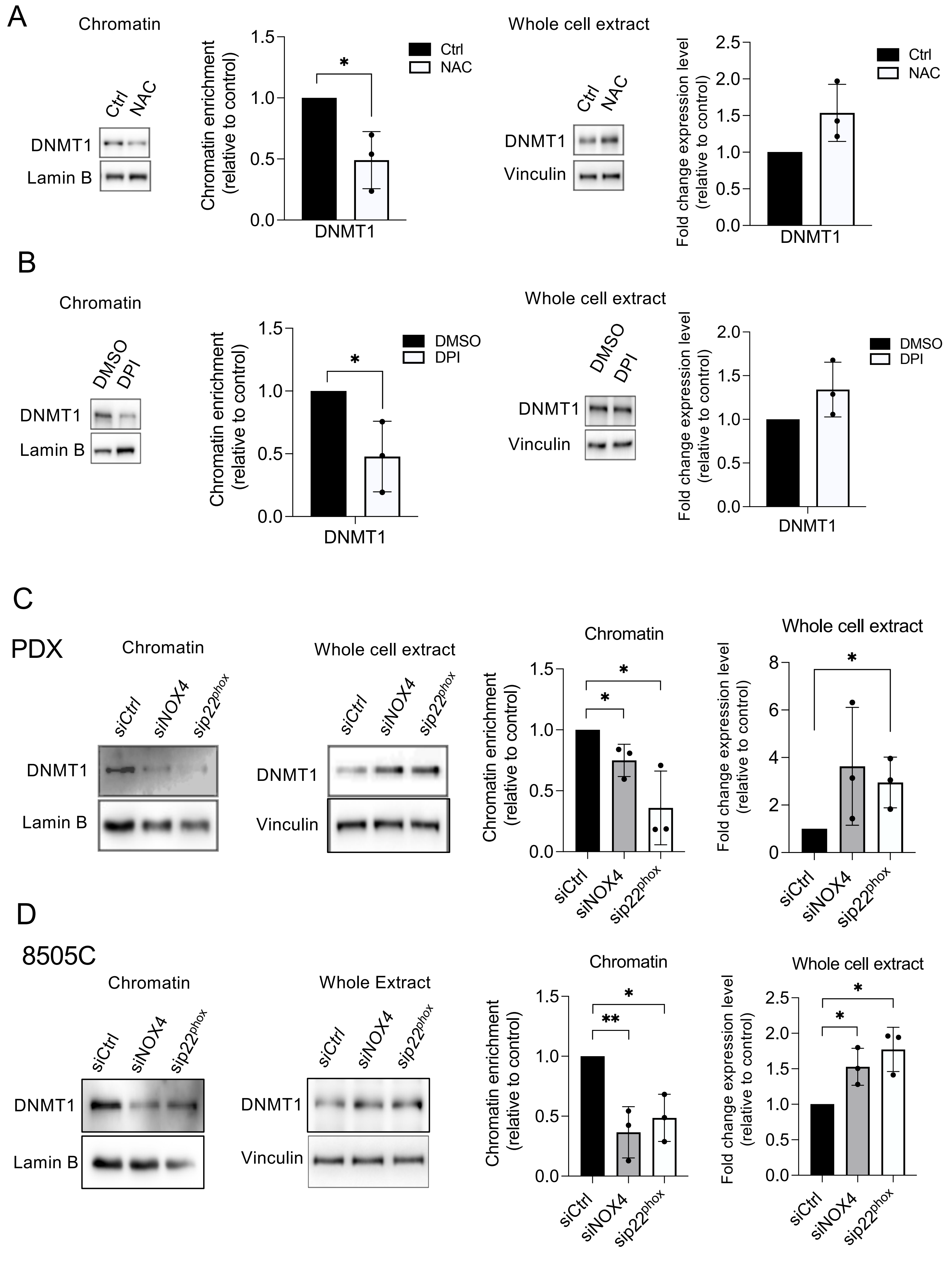

A
Chromatin
Ctrl
NAC
DNMT1
Lamin B
Chromatin enrichment (relative to control)
1.5
1.0
0.5
0.0
*
DNMT1
Ctrl
NAC
Whole cell extract
Ctrl
NAC
DNMT1
Vinculin
Fold change expression level (relative to control)
2.5
2.0
1.5
1.0
0.5
0.0
DNMT1
Ctrl
NAC
B
Chromatin
DMSO
DPI
DNMT1
Lamin B
Chromatin enrichment (relative to control)
*
DNMT1
DMSO
DPI
Whole cell extract
DMSO
DPI
DNMT1
Vinculin
Fold change expression level (relative to control)
DNMT1
DMSO
DPI
C
PDX
Chromatin
siCtrl
siNOX4
sip22phox
DNMT1
Lamin B
Whole cell extract
siCtrl
siNOX4
sip22phox
DNMT1
Vinculin
Chromatin
Chromatin enrichment (relative to control)
*
*
siCtrl
siNOX4
sip22phox
Whole cell extract
Fold change expression level (relative to control)
8
6
4
2
0
*
siCtrl
siNOX4
sip22phox
D
8505C
Chromatin
siCtrl
siNOX4
sip22phox
DNMT1
Lamin B
Whole Extract
siCtrl
siNOX4
sip22phox
DNMT1
Vinculin
Chromatin
Chromatin enrichment (relative to control)
**
*
siCtrl
siNOX4
sip22phox
Whole cell extract
Fold change expression level (relative to control)
*
*
siCtrl
siNOX4
sip22phox

Figure S6: Anti-oxidant (NAC) or NADPH oxidase inhibitor (DPI) alters the recruitment of DNMT1 to chromatin in BRAF-mutated thyroid cells. A) BCPAP cells were pre-treated with 5 mM NAC for 2 h before being analysed for DNMT1 protein expression level in chromatin fractions and whole-cell extracts. B) BCPAP cells were pre-treated with 1 μM DPI for 6 h before being analysed for DNMT1 protein expression level in chromatin fractions and whole-cell extracts. C) Western blot analysis of DNMT1 protein expression level in chromatin fractions and whole-cell extracts 72 h after knocking down of NOX4 or $p22^{phox}$ by RNA interference in PDX cells. D) Western blot analysis of DNMT1 protein expression level in chromatin fractions, and whole-cell extracts 72 h after knocking down of NOX4 or $p22^{phox}$ by RNA interference in 8505C cells. Densitometry quantification of protein levels normalized to Lamin B or Vinculin levels and presented as chromatin enrichment or fold change compared with siRNA control-transduced cells. Values are mean ± SE. $*p < 0.05$ and $**p < 0.01$ ($n = 3$).

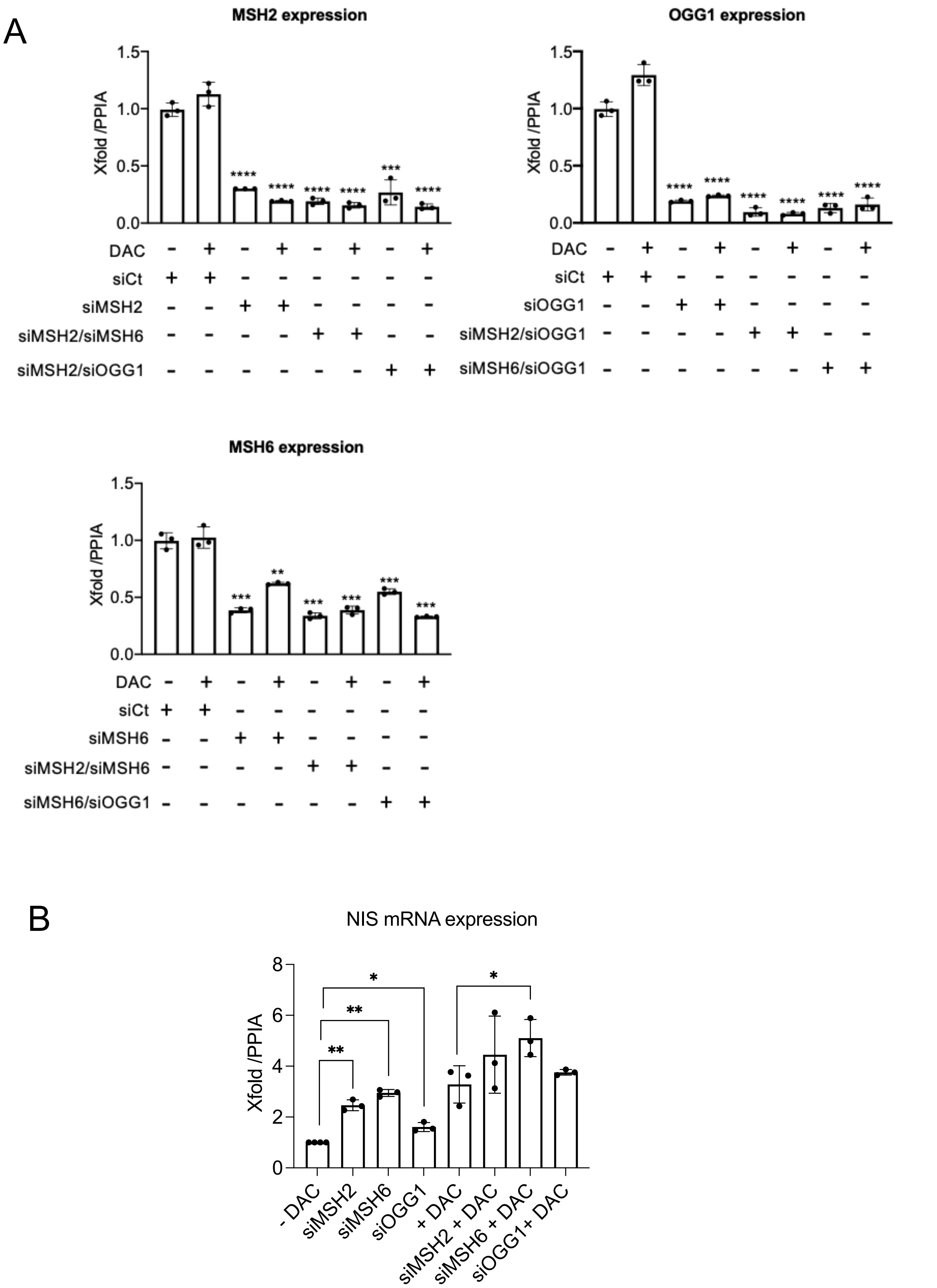

Figure S7: Validation of siRNA efficiency by qPCR (A) and analysis of the effect of single siRNA with or without decitabine (DAC) on NIS mRNA expression (B). A) qRT-PCR analysis of MSH2,MSH6 and OGG1 mRNA levels in BCPAP cells transfected with siRNA control or/and siRNA MSH2 or/and siRNA MSH6 or/and siRNA OGG1 and 24 h later treated for an additional 48 h in the presence or the absence of 1 µM DAC. B) qRT-PCR analysis of NIS mRNA levels in BCPAP cells transfected with siRNA control or siRNA MSH2 or siRNA MSH6 or siRNA OGG1 and 24 h later treated for an additional 48 h in the presence or the absence of 1 µM DAC. Values are mean ± SE.
*$p < 0.05$, **$p < 0.01$, ***$p < 0.001$ and ****$p<0.0001$ (n=3) .

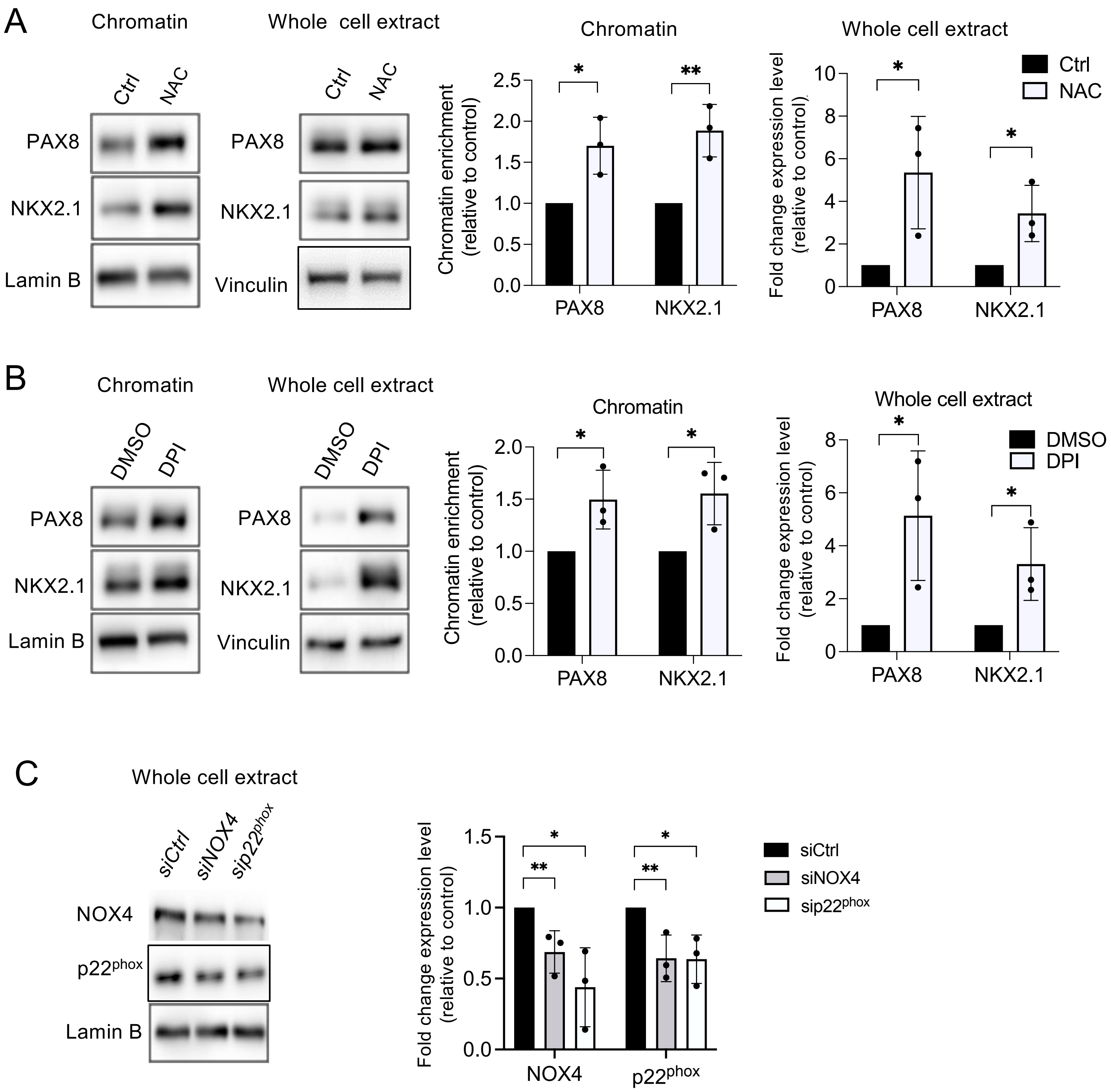

Figure S8: Antioxidants promote recruitment of PAX8 and NKX2.1 to chromatin**.** A) BCPAP cells were pre-treated with 5 mM NAC for 2 h before being analysed for PAX8 and NKX2.1 protein expression levels in chromatin fractions and whole-cell extracts. B) BCPAP cells were pre-treated with 1 µM DPI for 6 h before being analysed for PAX8 and NKX2.1 protein expression levels in chromatin fractions and whole-cell extracts. C) siRNA knockdown validation of NOX4 or $p22^{phox}$ protein expression levels analysed by Western-blot in whole-cell extracts 72h after knocking down of NOX4 or p22phox by RNA interference in BCPAP cells. Densitometry quantification of protein levels normalized to Lamin B or Vinculin levels and presented as chromatin enrichment or fold change compared with control cells. Values are mean ± SE. *p < 0.05 and **p < 0.01 (n = 3).

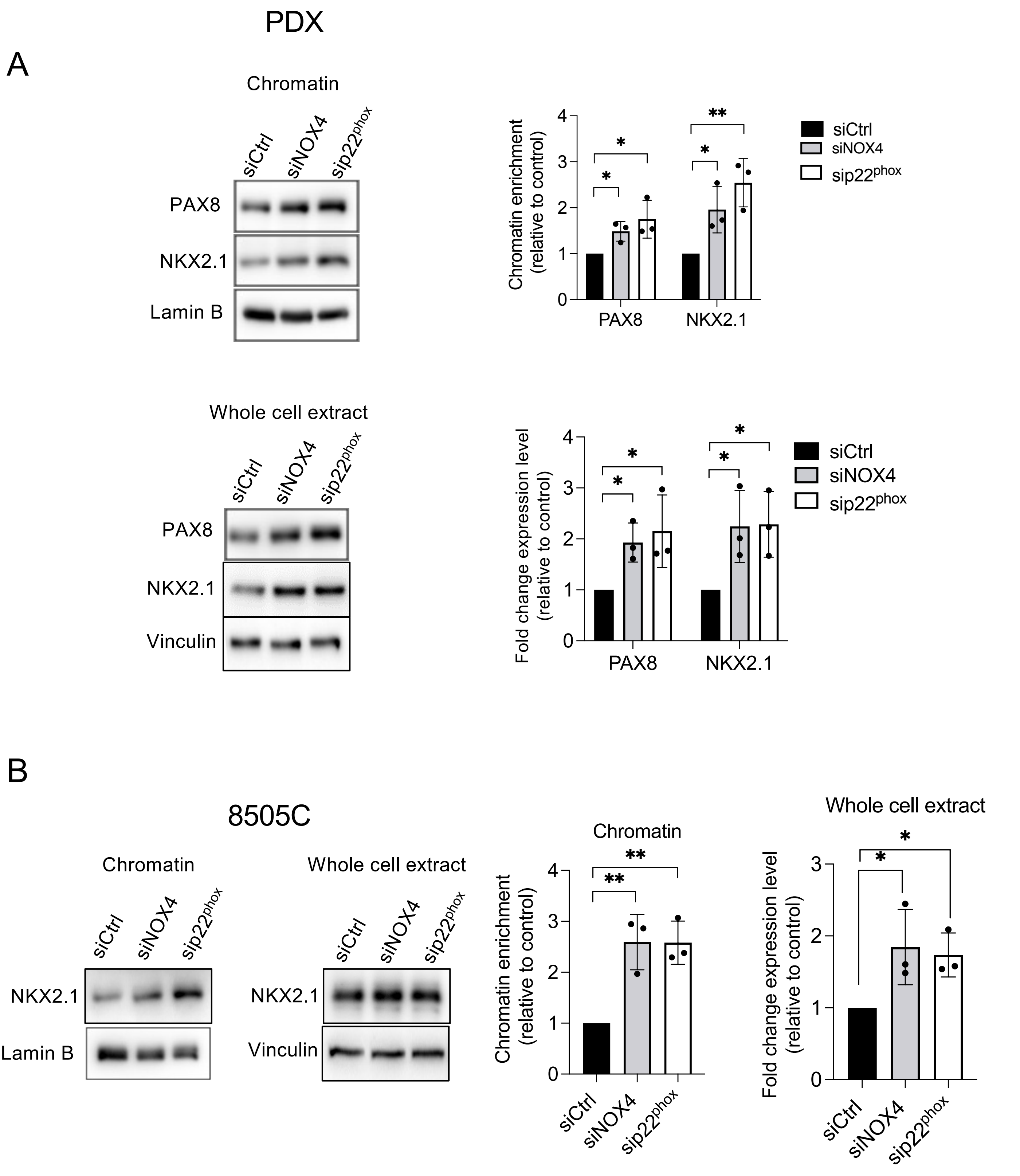

Figure S9: NOX4 inhibits recruitment of PAX8 and NKX2.1 to chromatin. A) Western blot analysis of PAX8 and NKX2.1 protein expression levels in chromatin fractions and whole-cell extracts 72 h after knocking down of NOX4 or p22phox by RNA interference in PDX cells. B) Western blot analysis of NKX2.1 protein expression levels in chromatin fractions and whole-cell extracts 72 h after knocking down of NOX4 or p22phox by RNA interference in 8505C cells. Densitometry quantification of protein levels normalized to Lamin B or Vinculin levels and presented as chromatin enrichment or fold change compared with control cells. Values are mean ± SE. *$p < 0.05$ and **$p < 0.01$ ($n = 3$).

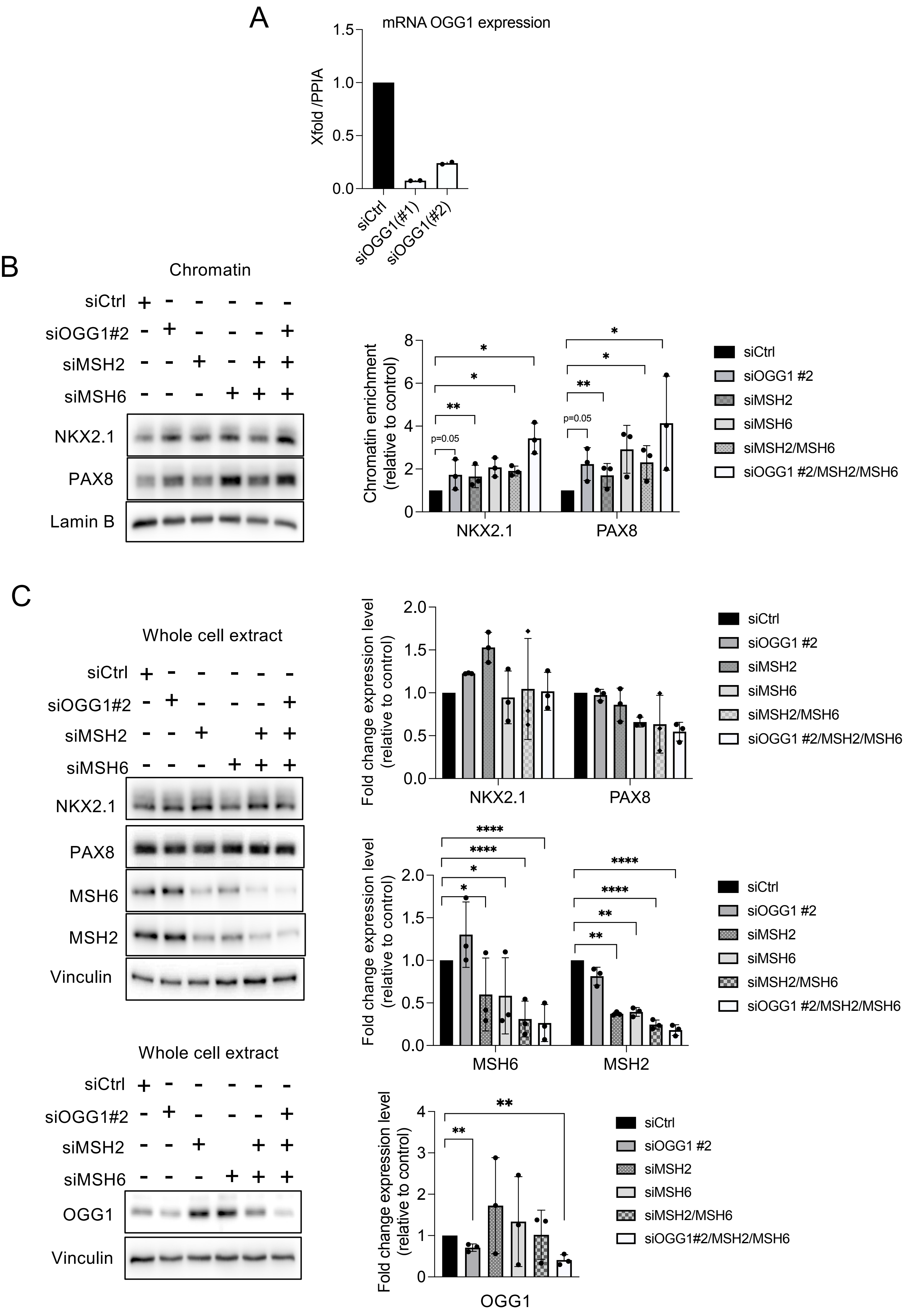
A
mRNA OGG1 expression
Xfold /PPIA
1.5
1.0
0.5
0.0
siCtrl
siOGG1(#1)
siOGG1(#2)
B
Chromatin
siCtrl
siOGG1#2
siMSH2
siMSH6
NKX2.1
PAX8
Lamin B
Chromatin enrichment
(relative to control)
p=0.05
NKX2.1
PAX8
siCtrl
siOGG1 #2
siMSH2
siMSH6
siMSH2/MSH6
siOGG1 #2/MSH2/MSH6
C
Whole cell extract
siCtrl
siOGG1#2
siMSH2
siMSH6
NKX2.1
PAX8
MSH6
MSH2
Vinculin
Fold change expression level
(relative to control)
NKX2.1
PAX8
siCtrl
siOGG1 #2
siMSH2
siMSH6
siMSH2/MSH6
siOGG1 #2/MSH2/MSH6
Fold change expression level
(relative to control)
MSH6
MSH2
Whole cell extract
siCtrl
siOGG1#2
siMSH2
siMSH6
OGG1
Vinculin
Fold change expression level
(relative to control)
OGG1
siCtrl
siOGG1 #2
siMSH2
siMSH6
siMSH2/MSH6
siOGG1#2/MSH2/MSH6

Figure S10: OGG1, MSH2, and MSH6 inhibit recruitment of PAX8 and NKX2.1 to chromatin in BCPAP cells. A) Validation of the siRNA transfection efficiency – Quantitative RT-PCR shows gene downregulation after 24h of transfection with two different siRNA targeting OGG1 (n=2). B) Western blot analysis of PAX8 and NKX2.1 protein expression levels in chromatin fractions 72 h after knocking down of OGG1 or MSH2 or MSH6 by RNA interference in BCPAP cells. C) Western blot analysis of PAX8, NKX2.1, MSH6, MSH2 and OGG1 protein expression levels in whole-cell extracts 72 h after knocking down of OGG1 or MSH2 and MSH6 by RNA interference in BCPAP cells. Densitometry quantification of protein levels normalized to Lamin B or Vinculin levels and presented as chromatin enrichment or fold change compared with control cells. Values are mean ± SE. *$p < 0.05$ and **$p < 0.01$ and ****$p<0.0001$ ($n = 3$).

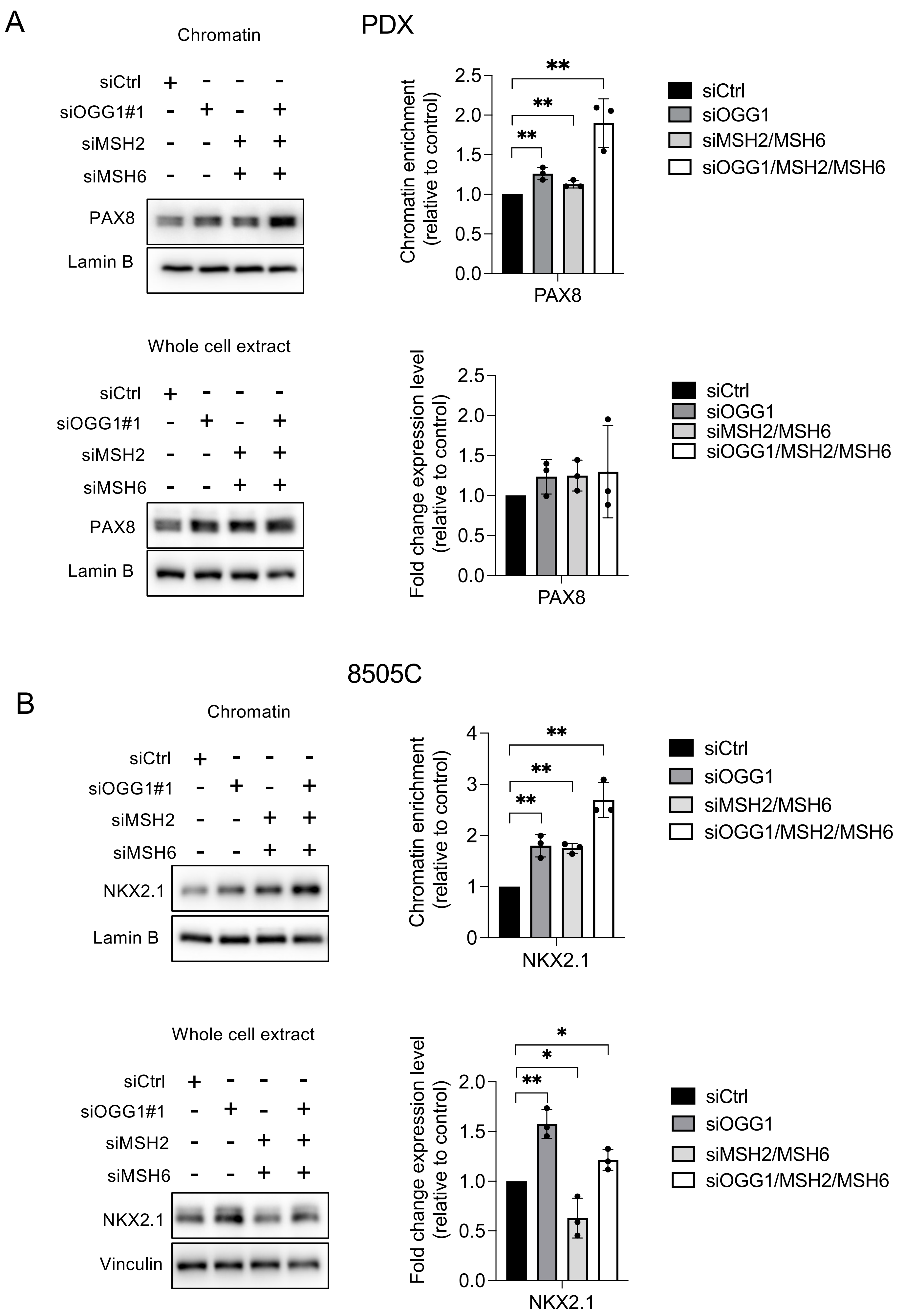
A
PDX
Chromatin
siCtrl + - - -
siOGG1#1 - + - +
siMSH2 - - + +
siMSH6 - - + +
PAX8
Lamin B
Chromatin enrichment (relative to control)
0.0
0.5
1.0
1.5
2.0
2.5
**
**
**
PAX8
siCtrl
siOGG1
siMSH2/MSH6
siOGG1/MSH2/MSH6
Whole cell extract
siCtrl + - - -
siOGG1#1 - + - +
siMSH2 - - + +
siMSH6 - - + +
PAX8
Lamin B
Fold change expression level (relative to control)
0.0
0.5
1.0
1.5
2.0
2.5
PAX8
siCtrl
siOGG1
siMSH2/MSH6
siOGG1/MSH2/MSH6
8505C
B
Chromatin
siCtrl + - - -
siOGG1#1 - + - +
siMSH2 - - + +
siMSH6 - - + +
NKX2.1
Lamin B
Chromatin enrichment (relative to control)
0
1
2
3
4
**
**
**
NKX2.1
siCtrl
siOGG1
siMSH2/MSH6
siOGG1/MSH2/MSH6
Whole cell extract
siCtrl + - - -
siOGG1#1 - + - +
siMSH2 - - + +
siMSH6 - - + +
NKX2.1
Vinculin
Fold change expression level (relative to control)
0.0
0.5
1.0
1.5
2.0
*
*
**
NKX2.1
siCtrl
siOGG1
siMSH2/MSH6
siOGG1/MSH2/MSH6

Figure S11: OGG1, MSH2, and MSH6 inhibit recruitment of PAX8 and NKX2.1 to chromatin in PDX and 8505C cells. A) Western blot analysis of PAX8 protein expression level in chromatin fractions and whole-cell extracts 72 h after knocking down of OGG1 or MSH2 and MSH6 by RNA interference in PDX cells. B) Western blot analysis of NKX2.1 protein expression level in chromatin fractions and whole-cell extracts 72 h after knocking down of OGG1 or MSH2 and MSH6 by RNA interference in 8505C cells. Densitometry quantification of protein levels normalized to Lamin B or Vinculin levels and presented as chromatin enrichment or fold change compared with control cells. Values are mean ± SE. $^{*}p < 0.05$ and $^{**}p < 0.01$ ($n = 3$).

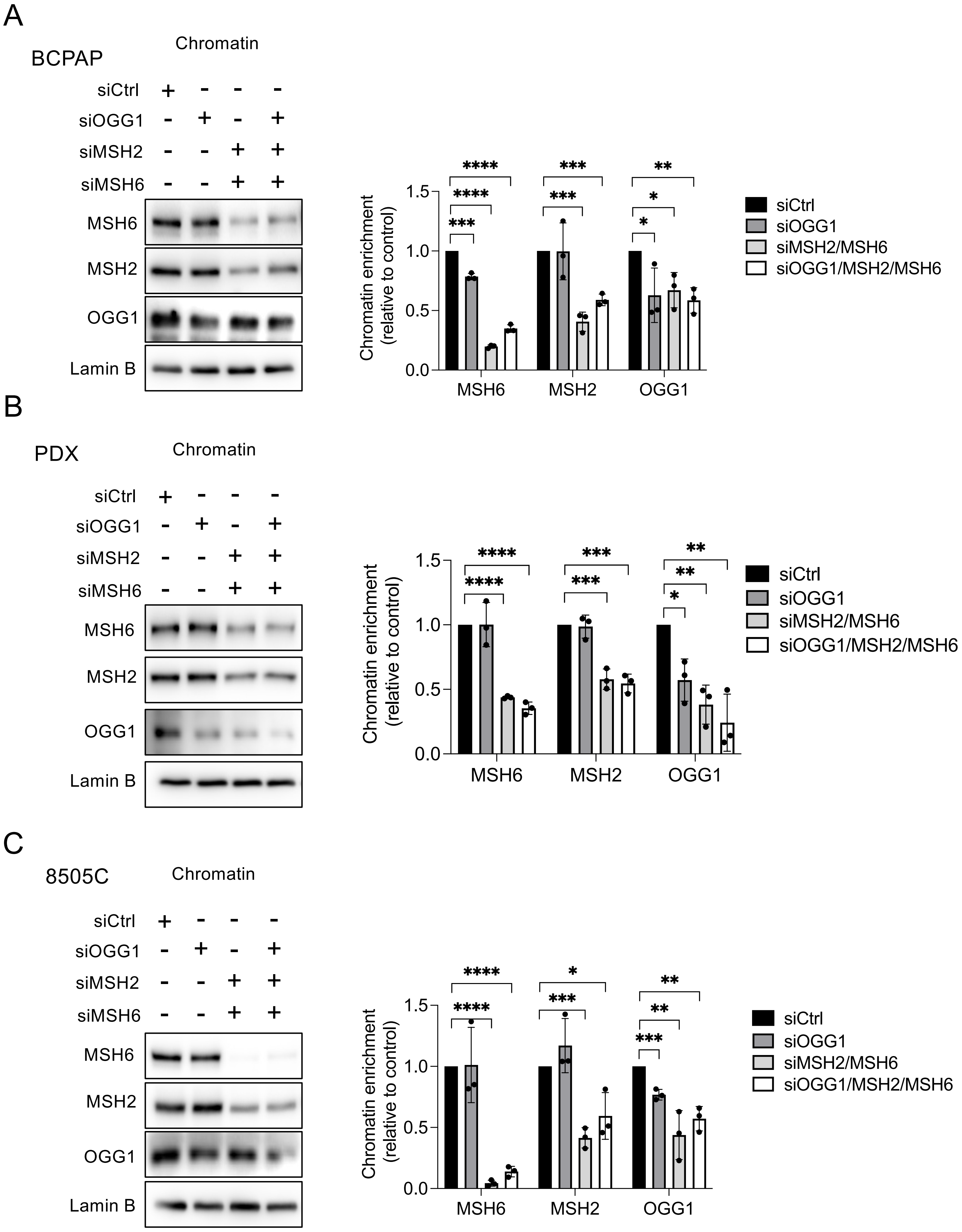
A
BCPAP
Chromatin
siCtrl + - - -
siOGG1 - + - +
siMSH2 - - + +
siMSH6 - - + +
MSH6
MSH2
OGG1
Lamin B
Chromatin enrichment (relative to control)
0.0
0.5
1.0
1.5
****
****
***
***
***
**
*
*
MSH6
MSH2
OGG1
siCtrl
siOGG1
siMSH2/MSH6
siOGG1/MSH2/MSH6
B
PDX
Chromatin
siCtrl + - - -
siOGG1 - + - +
siMSH2 - - + +
siMSH6 - - + +
MSH6
MSH2
OGG1
Lamin B
Chromatin enrichment (relative to control)
0.0
0.5
1.0
1.5
****
****
***
***
**
**
*
MSH6
MSH2
OGG1
siCtrl
siOGG1
siMSH2/MSH6
siOGG1/MSH2/MSH6
C
8505C
Chromatin
siCtrl + - - -
siOGG1 - + - +
siMSH2 - - + +
siMSH6 - - + +
MSH6
MSH2
OGG1
Lamin B
Chromatin enrichment (relative to control)
0.0
0.5
1.0
1.5
****
****
*
***
**
**
***
MSH6
MSH2
OGG1
siCtrl
siOGG1
siMSH2/MSH6
siOGG1/MSH2/MSH6

Figure S12: siRNA knockdown validation of OGG1, MSH2, and MSH6 analysed in chromatin fraction of BRAF-mutated thyroid cells. A) Western blot analysis of OGG1, MSH2, and MSH6 protein expression levels in chromatin fractions 72 h after knocking down of OGG1, MSH2, and MSH6 in BCPAP cells. B) Western blot analysis of OGG1, MSH2, and MSH6 protein expression levels in chromatin fractions 72 h after knocking down of OGG1, MSH2, and MSH6 in PDX cells. C) Western blot analysis of OGG1, MSH2, and MSH6 protein expression levels in chromatin fractions 72 h after knocking down of OGG1, MSH2, and MSH6 in 8505C cells. Densitometry quantification of protein levels normalized to Lamin B levels and presented as chromatin enrichment compared with control cells. Values are mean ± SE. *$p < 0.05$, **$p < 0.01$, ***$p < 0.001$ and ****$p<0.0001$ ($n = 3$).

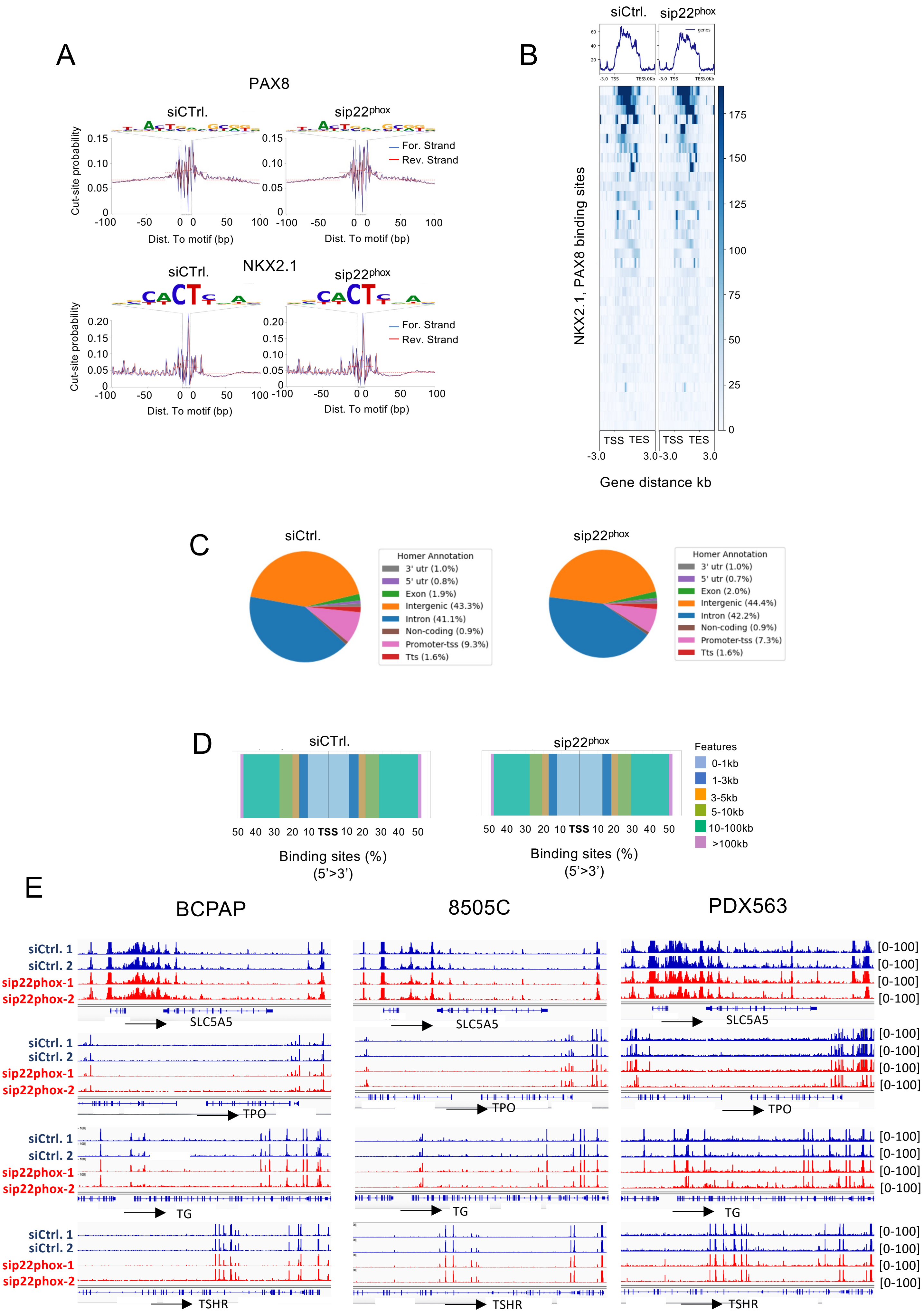
A
PAX8
siCTrl.
sip22phox
Cut-site probability
Dist. To motif (bp)
For. Strand
Rev. Strand
NKX2.1
B
siCtrl.
sip22phox
NKX2.1, PAX8 binding sites
TSS TES
Gene distance kb
C
siCtrl.
Homer Annotation
3' utr (1.0%)
5' utr (0.8%)
Exon (1.9%)
Intergenic (43.3%)
Intron (41.1%)
Non-coding (0.9%)
Promoter-tss (9.3%)
Tts (1.6%)
sip22phox
Homer Annotation
3' utr (1.0%)
5' utr (0.7%)
Exon (2.0%)
Intergenic (44.4%)
Intron (42.2%)
Non-coding (0.9%)
Promoter-tss (7.3%)
Tts (1.6%)
D
siCTrl.
sip22phox
Binding sites (%)
(5'>3')
Features
0-1kb
1-3kb
3-5kb
5-10kb
10-100kb
>100kb
E
BCPAP
8505C
PDX563
siCtrl. 1
siCtrl. 2
sip22phox-1
sip22phox-2
[0-100]
SLC5A5
TPO
TG
TSHR

Figure S13: ATAC-seq analysis of BRAF-mutated thyroid cells deleted or not for p22^{phox}. A) ATAC-seq footprint at the PAX8 and NKX2.1 full sites. Cut sites probability, insertions per site are normalized to have the same average depth of insertions ± 100 bp away from motif center. The DNA sequences of the bottom are the motif of PAX8 and NKX2.1, respectively. B) Tornado plots of ATAC seq signals representing sites around the NKX2.1 and PAX8 genes (±3 kb center). C) Genomic distribution of ATAC-seq peaks in BCPAP cells transduced for 72 h with siRNA control and siRNA p22^{phox}. D) Distribution of transcription factor-binding loci relative to TSS. E) Representative sequencing tracks for the *SLC5A5, TPO, TG,* and *TSHR* loci show distinct ATAC-seq peaks. The ATAC-seq data have been normalized to take sequencing depth into account and the scale on the y-axis was chosen for optimal visualization of peaks for each sample (n = 2).

8505C

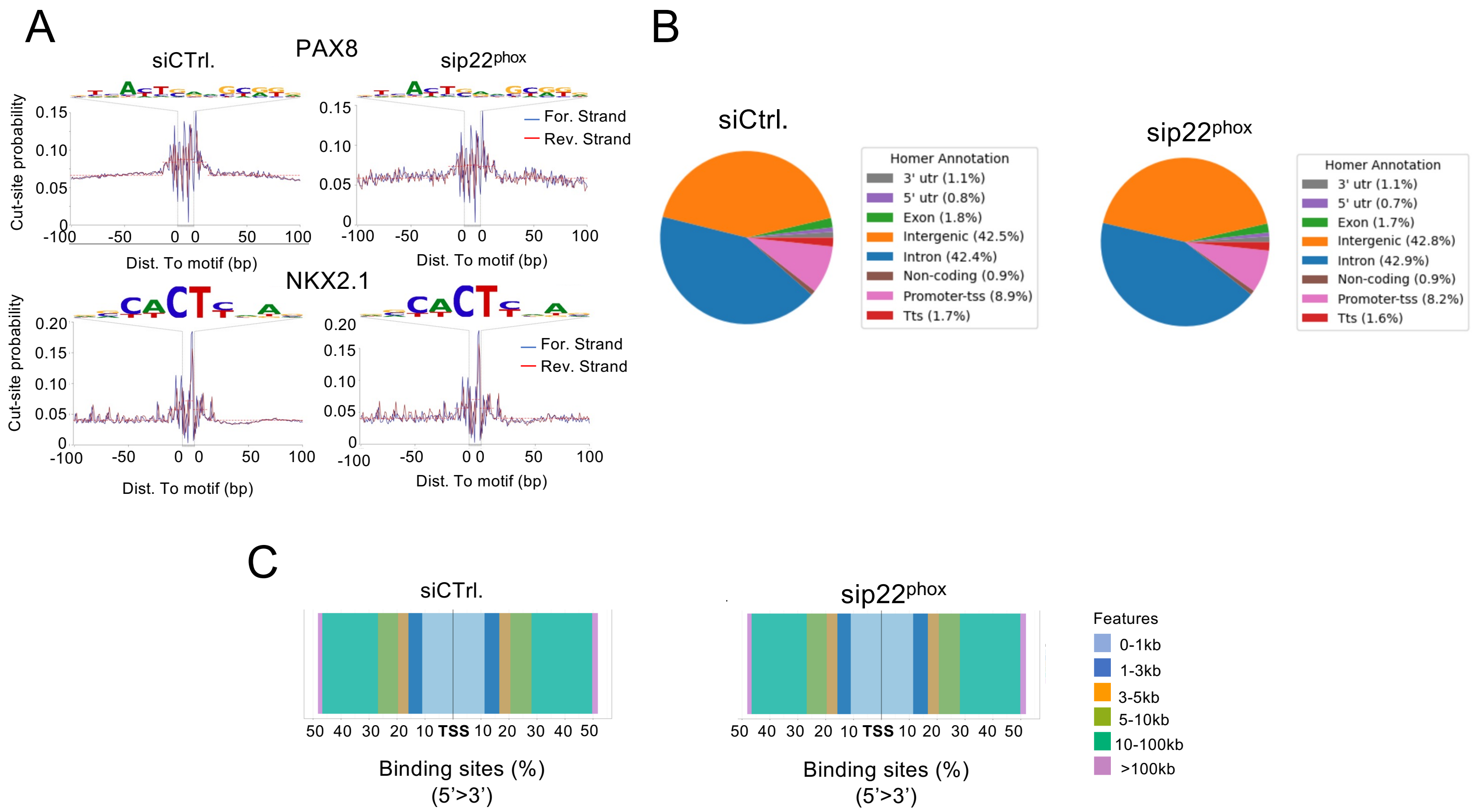
A
PAX8
siCTrl.
sip22phox
Cut-site probability
For. Strand
Rev. Strand
Dist. To motif (bp)
NKX2.1
B
siCtrl.
sip22phox
Homer Annotation
3' utr (1.1%)
5' utr (0.8%)
Exon (1.8%)
Intergenic (42.5%)
Intron (42.4%)
Non-coding (0.9%)
Promoter-tss (8.9%)
Tts (1.7%)
Homer Annotation
3' utr (1.1%)
5' utr (0.7%)
Exon (1.7%)
Intergenic (42.8%)
Intron (42.9%)
Non-coding (0.9%)
Promoter-tss (8.2%)
Tts (1.6%)
C
siCTrl.
sip22phox
50 40 30 20 10 TSS 10 20 30 40 50
Binding sites (%)
(5'>3')
Features
0-1kb
1-3kb
3-5kb
5-10kb
10-100kb
>100kb

PDX

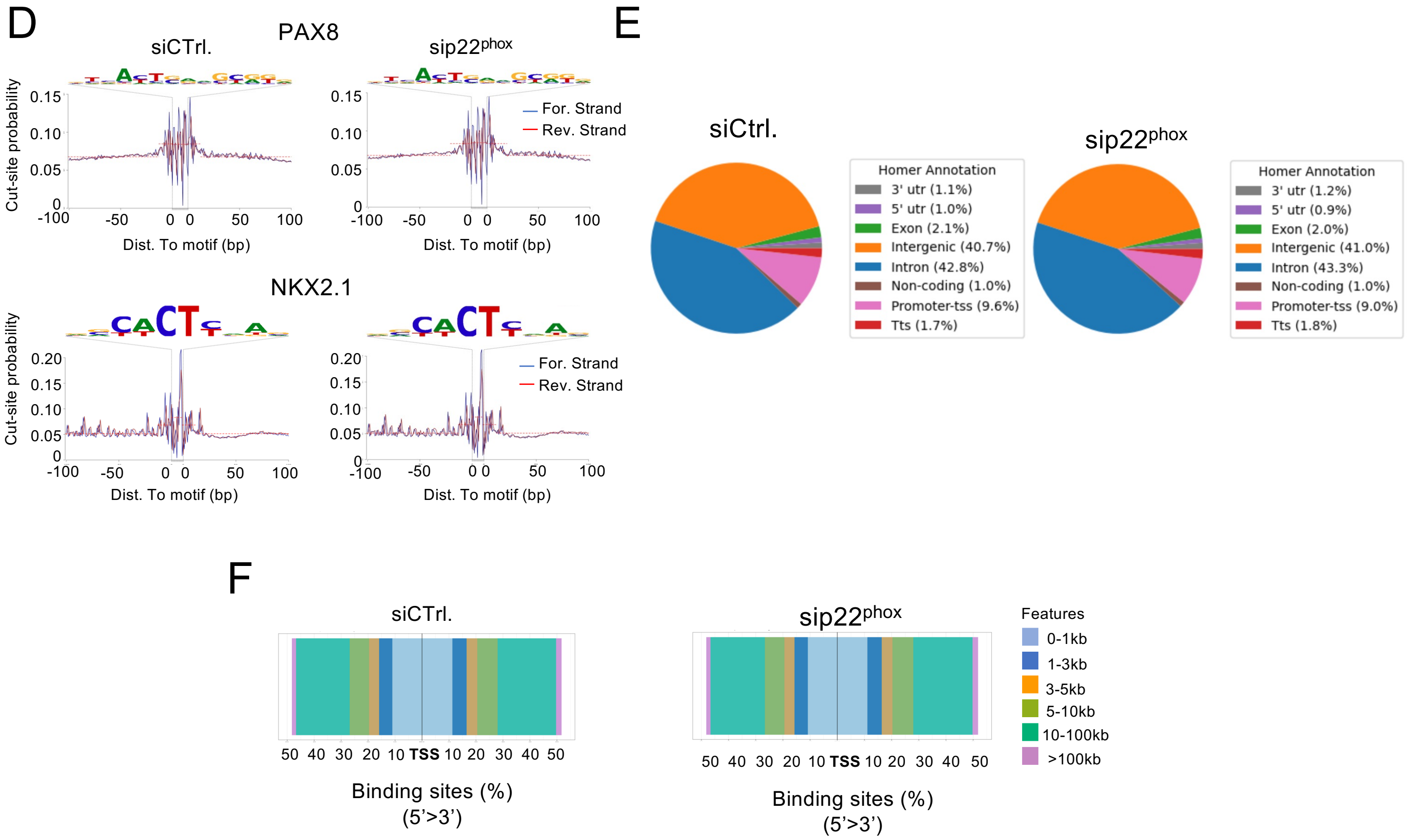
D
PAX8
siCTrl.
sip22phox
Cut-site probability
For. Strand
Rev. Strand
Dist. To motif (bp)
NKX2.1
E
siCtrl.
sip22phox
Homer Annotation
3' utr (1.1%)
5' utr (1.0%)
Exon (2.1%)
Intergenic (40.7%)
Intron (42.8%)
Non-coding (1.0%)
Promoter-tss (9.6%)
Tts (1.7%)
Homer Annotation
3' utr (1.2%)
5' utr (0.9%)
Exon (2.0%)
Intergenic (41.0%)
Intron (43.3%)
Non-coding (1.0%)
Promoter-tss (9.0%)
Tts (1.8%)
F
siCTrl.
sip22phox
50 40 30 20 10 TSS 10 20 30 40 50
Binding sites (%)
(5'>3')
Features
0-1kb
1-3kb
3-5kb
5-10kb
10-100kb
>100kb

Figure S14: ATAC-seq analysis of 8505C and PDX cells deleted or not for $p22^{phox}$. A, D) ATAC-seq footprint at the PAX8 and NKX2.1 full sites. Cut sites probability, insertions per site are normalized to have the same average depth of insertions ± 100 bp away from motif center. The DNA sequences of the bottom are the motif of PAX8 and NKX2.1, respectively. B, E) Genomic distribution of ATAC-seq peaks in BCPAP cells transduced for 72 h with siRNA control and siRNA $p22^{phox}$. C, F) Distribution of transcription factor-binding loci relative to TSS (n = 2).

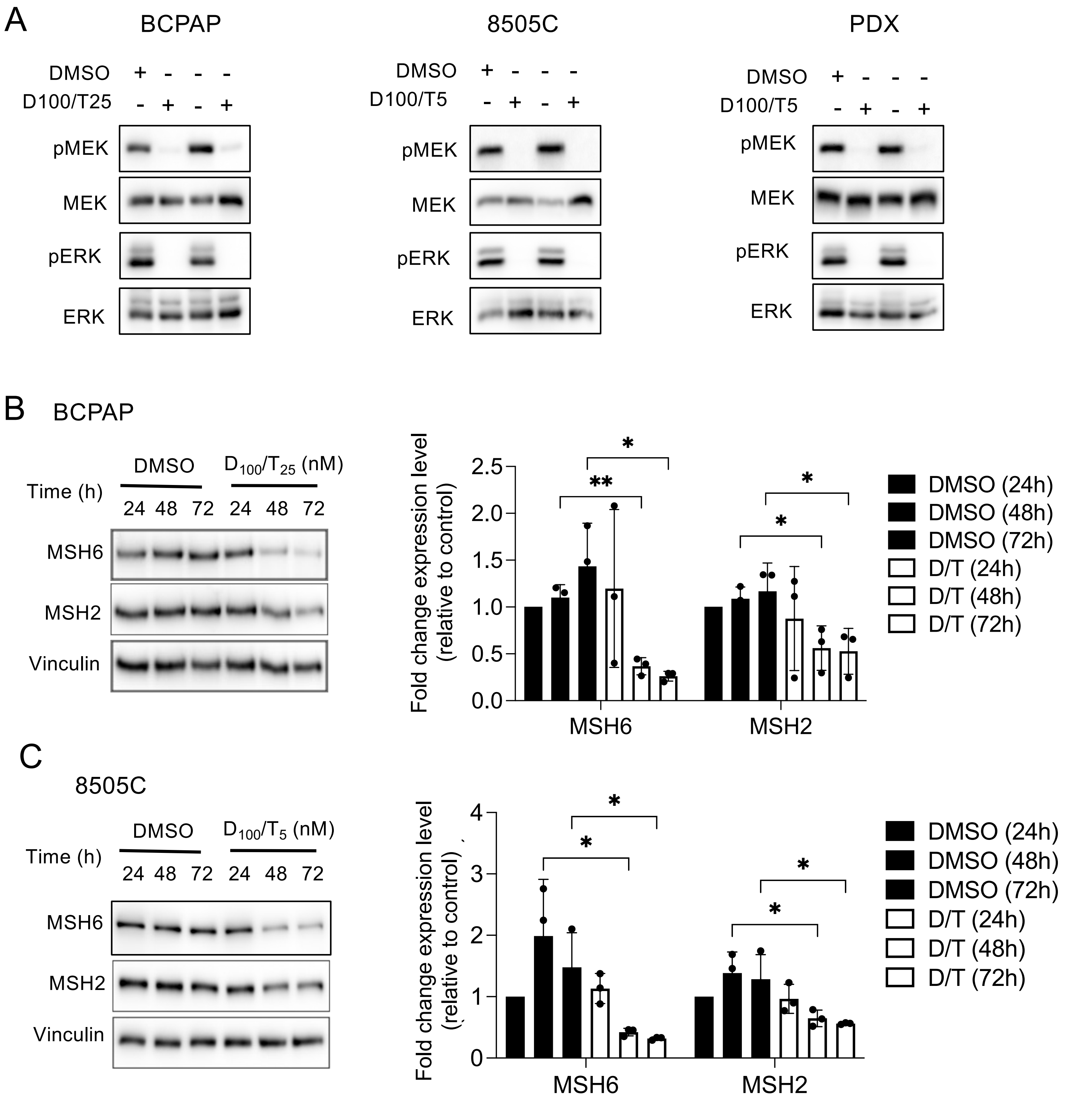
A
BCPAP
8505C
PDX
DMSO + - - -
D100/T25 - + - +
D100/T5 - + - +
pMEK
MEK
pERK
ERK
B
BCPAP
DMSO
D100/T25 (nM)
Time (h)
24 48 72 24 48 72
MSH6
MSH2
Vinculin
Fold change expression level (relative to control)
2.5
2.0
1.5
1.0
0.5
0.0
*
**
MSH6
MSH2
DMSO (24h)
DMSO (48h)
DMSO (72h)
D/T (24h)
D/T (48h)
D/T (72h)
C
8505C
D100/T5 (nM)
4
3
2
1
0

Figure S15: $BRAF^{V600E}$ regulates MSH2 and MSH6 expressions. A) BRAF-mutated thyroid cells were treated with Dabrafenib (100 nM) plus Trametinib (25 nM or 5 nM) for 4 h and analysed by Western-blot for expression level of pMEK, MEK, pERK, ERK and pERK. B) Immunoblot detection of MSH2 and MSH6 in PDX cells after treatment by the combination of Dabrafenib plus Trametinib. C) Immunoblot detection of MSH2 and MSH6 in 8505C cells after treatment by the combination of Dabrafenib plus Trametinib. Densitometric quantification of protein levels normalized to vinculin levels and presented as fold change compared with vehicle-treated cells. The Student t-test was done by comparing combination versus DMSO for each corresponding time of kinetic. Values are mean ± SE. $*p < 0.05$ and $**p < 0.01$ ($n = 3$).

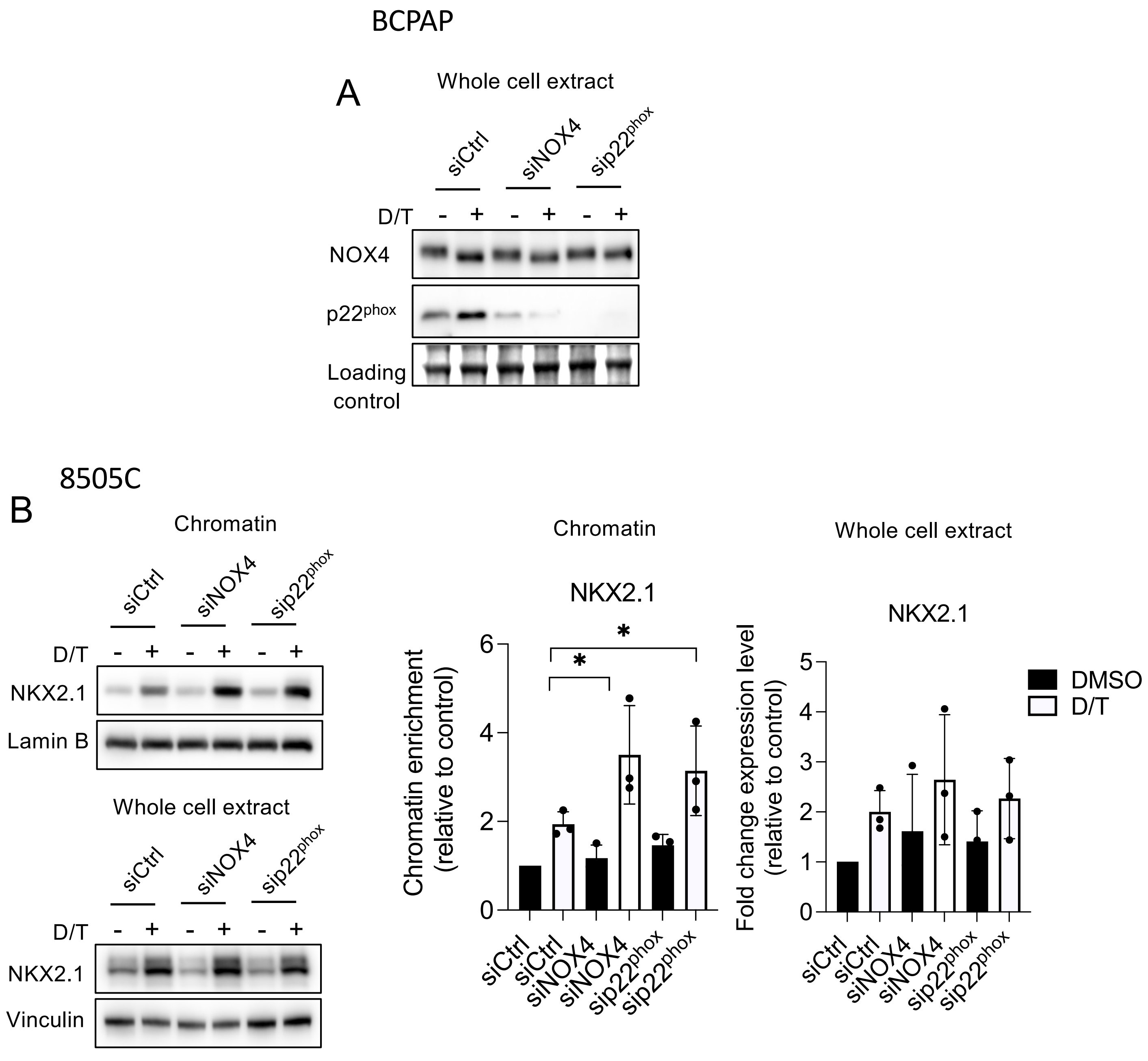

Figure S16: MAPK pathway inhibition and knockdown of NOX4 and p22^{phox} synergize to promote PAX8 and NKX2.1 recruitment to chromatin. A) siRNA knockdown validation of NOX4 and p22^{phox} protein expression levels analysed by Western-blot in whole-cell extract 72 h after knocking down of NOX4 or p22^{phox} by RNA interference in BCPAP cells. B) Western-blot analysis of NKX2.1 protein expression levels in chromatin fractions and whole-cell extracts of 8505C cells transduced with siRNA control or siRNA NOX4 or siRNA p22^{phox} and treated with Dabrafenib plus Trametinib combination for 48 h. Densitometric quantification of protein levels normalized to loading control and presented as fold change compared with siRNA control-transduced cells. Values are mean ± SE. *$p < 0.05$ ($n = 3$).

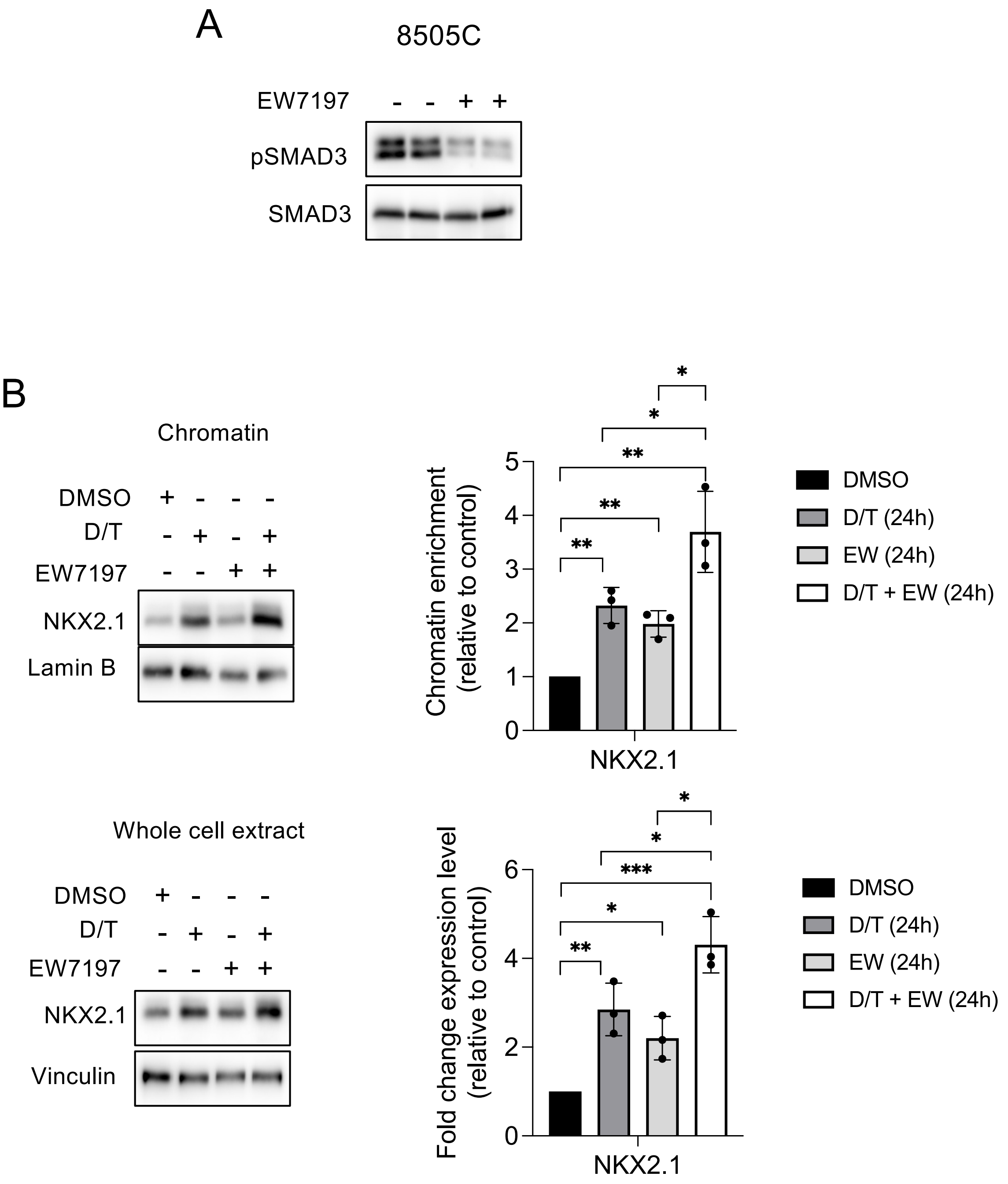

Figure S17: Co-inhibition of SMAD and MAPK signalling promotes NKX2.1 recruitment to chromatin. A) BRAF-mutated thyroid cells were treated with TGF-beta receptor inhibitor (EW7197, 1 µM) for 4 h and analysed by Western-blot for expression of pSMAD3 and SMAD3. B) Western-blot analysis of NKX2.1 protein expression level in chromatin fractions and whole-cell extracts of 8505C cells treated or not with Dabrafenib plus Trametinib combination in the presence or the absence of EW7197 for 24 h. Densitometry quantification of protein levels normalized to Lamin B or Vinculin levels and presented as chromatin enrichment or fold change compared with control cells. Values are mean ± SE. *p < 0.05, **p < 0.01, and ***p < 0.001 (n=3).

Table S1 : Details of samples available for Immunohistochemistry analysis

Sample ID	BRAF mut.	Tumor cell %	Tissu Type
05-01	p.V600E	80	Primitive Tumor
04-16	p.V600E	90	Lymph Node
14-36	p.V600E	90	Metastasis
11-33	p.V600E	90	Primitive Tumor
02-02	p.V600E	90	Primitive Tumor
14-22	p.V600E	80	Metastasis
06-06	p.V600E	80	Primitive Tumor
01-23	p.V600E	90	Metastasis
14-37	p.V600E	90	Primitive Tumor
01-13	p.V600E	80	Primitive Tumor
06-28	p.V600E	90	Primitive Tumor
03-14	p.V600E	90	Primitive Tumor
01-35	p.V600E	90	Primitive Tumor
01-39	p.V600E	80	Primitive Tumor
03-15	p.V600E	>90	Metastasis
